# Global soil metagenomics reveals distribution and predominance of *Deltaproteobacteria* in nitrogen-fixing microbiome

**DOI:** 10.1186/s40168-024-01812-1

**Published:** 2024-05-24

**Authors:** Yoko Masuda, Kazumori Mise, Zhenxing Xu, Zhengcheng Zhang, Yutaka Shiratori, Keishi Senoo, Hideomi Itoh

**Affiliations:** 1 Department of Applied Biological Chemistry, Graduate School of Agricultural and Life Sciences, The University of Tokyo, 1-1-1 Yayoi, Bunkyo-ku, Tokyo, 113-8657 Japan; 2 Collaborative Research Institute for Innovative Microbiology, The University of Tokyo, 1-1-1 Yayoi, Bunkyo-ku, Tokyo, 113-8657 Japan; 3 National Institute of Advanced Industrial Science and Technology (AIST) Hokkaido, 2-17-2-1 Tsukisamu-higashi, Toyohira, Sapporo, Hokkaido 062-8517 Japan; 4 Niigata Agricultural Research Institute, 857 Nagakura-machi, Nagaoka, Niigata, 940-0826 Japan

**Keywords:** microbial community, soil microbiome, metagenomics, nitrogen fixation

## Abstract

**Background:**

Biological nitrogen fixation is a fundamental process sustaining all life on earth. While distribution and diversity of N_2_-fixing soil microbes have been investigated by numerous PCR amplicon sequencing of nitrogenase genes, their comprehensive understanding has been hindered by lack of *de facto* standard protocols for amplicon surveys and possible PCR biases. Here, by fully leveraging the planetary collections of soil shotgun metagenomes along with recently expanded culture collections, we evaluated the global distribution and diversity of terrestrial diazotrophic microbiome.

**Results:**

After the extensive analysis of 1,451 soil metagenomic samples, we revealed that the *Anaeromyxobacteraceae* and *Geobacteraceae* within *Deltaproteobacteria* are ubiquitous groups of diazotrophic microbiome in the soils with different geographic origins and land usage types, with particular predominance in anaerobic soils (paddy soils and sediments).

**Conclusion:**

Our results indicate that *Deltaproteobacteria* is a core bacterial taxon in the potential soil nitrogen fixation population, especially in anaerobic environments, which encourages a careful consideration on deltaproteobacterial diazotrophs in understanding terrestrial nitrogen cycling.

Video Abstract

**Supplementary Information:**

The online version contains supplementary material available at 10.1186/s40168-024-01812-1.

## Introduction

Biological nitrogen fixation driven by diverse soil microorganisms is a distinct process providing the pedosphere with nitrogen, the major limiting factor for primary production [1]. Microbial players for nitrogen fixation (diazotrophs) in the soil have drawn significant attention since their discovery in the late nineteenth century [2]. In particular, the distribution and diversity of the diazotrophs in the soil have been one of the most active research topics and is constantly updated along with the accumulation of knowledge and technological innovations [3–5].

Although nitrogenase genes (*nif*) are conserved in a broad taxonomic range of prokaryotes [6], *nif* genes derived from *Alphaproteobacteria*, *Betaproteobacteria*, and *Cyanobacteria* have been frequently detected in various soil environments such as farmland, grassland, forests, rice paddy fields, riparian zones, and tundra by PCR amplicon surveys targeting *nif* genes [7–11]. Consequently, these bacteria are considered the primary nitrogen fixers in soil [12, 13]. In our previous work, however, shotgun metagenomic and metatranscriptomic analyses of paddy soil at one site in Japan have detected highly abundant *nif* genes and transcripts from the families *Anaeromyxobacteraceae* and *Geobacteraceae* within *Deltaproteobacteria* (also classified as the phyla *Myxococcota* and *Desulfobacterota,* respectively) compared with the conventional diazotrophic groups [14]. Several amplicon-based studies have also reported the occurrence of *Geobacteraceae* nitrogenase genes in soils [15–18]. Considering their prevalence across many soil types as revealed by 16S rRNA gene-based surveys [19–21], members of the families *Anaeromyxobacteraceae* and *Geobacteraceae* may thus represent universal and/or major components of diazotrophic microbiome in various terrestrial environments. However, these clades, which are well-known iron-reducing bacterial groups [22], have received considerably less attention as diazotrophs in soil than the conventional groups.

One potential problem is that genomic information of *Anaeromyxobacteraceae* and *Geobacteraceae* has been poorly represented in reference databases because pure isolates of these bacteria have been difficult to obtain. Fortunately, recent studies significantly enriched the reference sequence databases by isolating dozens of novel members within these families using our previously developed slurry incubation method; preincubated soil slurry and Reasoner’s 2A (R2A) agar supplemented with fumarate were used as isolation source and medium, respectively, as described in details in Materials and methods section [23–31]. All of the novel isolates harbor nitrogenase genes, whereas some of them have been shown to present diazotrophic activities [25, 26, 29, 30].

Another problem is that amplicon sequencing tend to incur major biases in microbiome studies. Universal primers often fail to detect (even dominant) genes due to mismatches or abnormal GC contents of template DNA, whereas they may amplify homologous but unrelated genes [32–37]. Moreover, the primer sets (and accordingly PCR conditions) used for nitrogenase gene (typically *nifH*) amplification are not standardized among a plethora of amplicon sequencing studies [38]. This suggests that different studies bear different types and degrees of PCR biases. These call for extensive analyses of shotgun metagenomic data, rather than amplicon-based data, to establish minimally biased knowledge on diazotrophic communities in terrestrial microbiomes.

In this study, we aimed to re-evaluate the global distribution and diversity of the terrestrial diazotrophic microbiome considering the presence of *Anaeromyxobacteraceae* and *Geobacteraceae* bacteria. We scrutinized a global trend of the terrestrial diazotrophic microbiome using 1,451 shotgun metagenomic datasets, making full use of recently published genomic information on *Anaeromyxobacteraceae* and *Geobacteraceae* isolates. Our analyses revealed that *Anaeromyxobacteraceae* and *Geobacteraceae* are ubiquitous constituents of the diazotrophic microbiome in terrestrial ecosystems, particularly with high dominance in anaerobic environments.

## Results and discussion

### Diversity of *nif*-harboring genomes in public databases

**Fig. 1:**
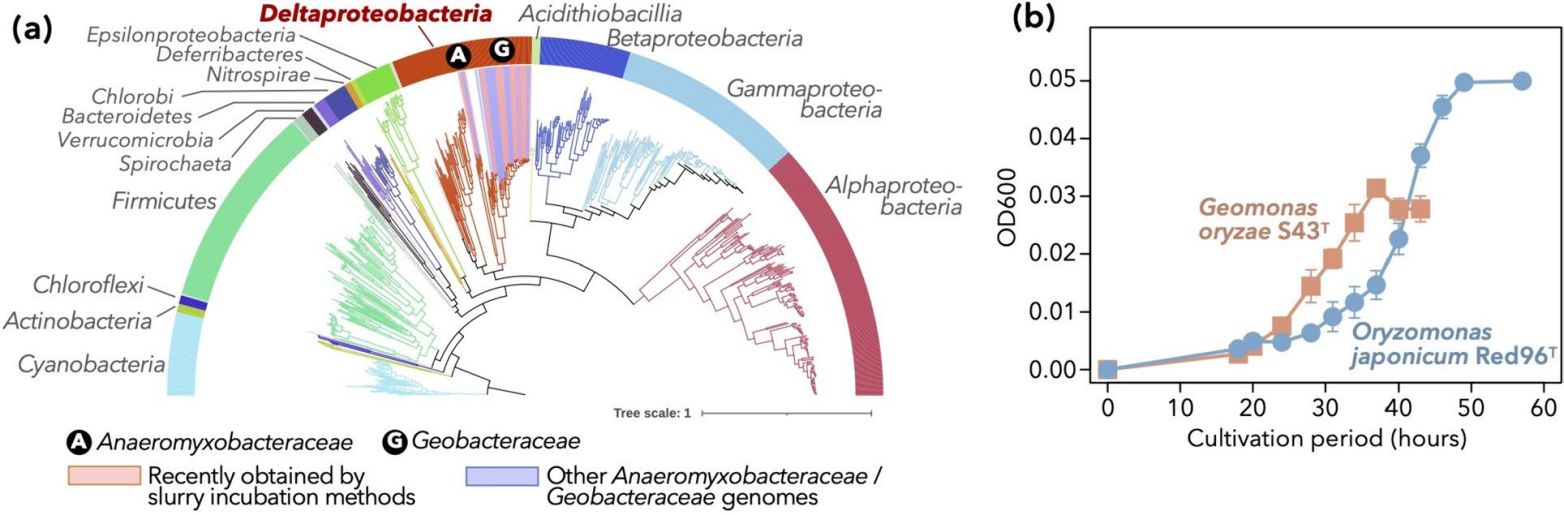
Currently known diversity of diazotrophs. **a** A genome-based phylogenetic tree consisting of potential diazotrophic bacteria [i.e., the genomes of which harbor all three core genes of nitrogenase (*nifH*, *nifD*, and *nifK*)], including the genomes of new isolates of *Anaeromyxobacteraceae* and *Geobacteraceae* (Table 1). The colors of branches and the band surrounding the tree denote the phyla and proteobacterial classes. Genomes of families *Anaeromyxobacteraceae* and *Geobacteraceae*, the foci of the present study, are highlighted with circled letters (A and G) and colored backgrounds (blue and pink, respectively). **b** Growth curves of the type strains of two type species within the family *Geobacteraceae*, namely *Geomonas oryzae* S43^T^ and *Oryzomonas japonica* Red96^T^. The two isolates were grown on MFM medium with N_2_ as the sole nitrogen source. Average and standard deviation of each time point (n = 3) are indicated. Some error bars are shorter than the symbol size

We first reviewed the currently known diversity of nitrogen-fixing prokaryotes in public databases. KEGG included 7,152 bacterial genomes (KEGG ftp as of August 31, 2022), and 697 of them encoded all three structural genes of nitrogenase, namely *nifH*, *nifD*, and *nifK*. Among these genomes, those of *Alphaproteobacteria*, *Firmicutes*, and *Gammaproteobacteria*, as well as *Deltaproteobacteria*, were abundant (Fig. 1a).

The representations of *Geobacteraceae* and *Anaeromyxobacteraceae* sequences in public databases were recently improved. At the end of 2018, RefSeq contained 23 genomes from *Geobacteraceae* and 5 from *Anaeromyxobacteraceae*, whereas these numbers tripled by September 28, 2022. Approximately 50% of these increases could be attributed to isolates obtained by the slurry incubation method since 2019 (Table 1) [23–31], and all of these isolates bear the core *nif* genes (*nifHDK*) in their genomes. Apart from these isolates, we obtained two other distinct strains belonging to the genus *Geomonas*, namely Red32 (isolated from paddy soil in Joetsu, Niigata, Japan) and Red276 (pond sediment in Myoko, Niigata, Japan; Table 1). These strains displayed 96.3%–97.4% similarity (based on 16S rRNA gene sequences) to all *Geomonas* type strains, which was below the standard threshold (98.65%) for species delineation [39]. The genomes of these strains also encoded *nifHDK*.

**Table 1:** Novel bacterial members within the families *Anaeromyxobacteraceae* and *Geobacteraceae* that have been recently isolated using a slurry incubation method and published to date, including ones isolated in this study. Relevant publications, as well as genomic information, are also indicated

Strain	Accession No. of genomic data	Note	Reference
*Anaeromyxobacteraceae*			
*Anaeromyxobacter diazotrophicus* Red267^T^	GCF_013340205.1		[24, 26]
*Anaeromyxobacter oryzae* Red232^T^	GCF_023169945.1		[24]
*Anaeromyxobacter paludicola* Red630^T^	GCF_023169965.1		[24]
*Geobacteraceae*			
*Geomonas azotofigens* Red51^T^	GCF_018919395.1		[29]
*Geomonas diazotrophica* Red69^T^	GCF_018919385.1		[29]
*Geomesophilobacter sediminis* Red875^T a^	GCF_016458275.1		[31]
*Geomonas propionica* Red259^T^	GCF_016458235.1		[31]
*Geomonas anaerohicana* Red421^T^	GCF_016458305.1		[31]
*Geomonas silvestris* Red330^T^	GCF_014193515.1		[23]
*Geomonas paludis* Red736^T^	GCF_014193585.1	GCF_023221575.1 is included in KEGG	[23]
*Geomonas paludis* RG22	GCF_023221575.1		[30]
*Geomonas limicola* Red745^T^	GCF_014193675.1		[23]
*Oryzomonas japonica* Red96^T a^	GCF_008802365.1		[27]
*Oryzomonas sagensis* Red100^T^	GCF_008802355.1		[27]
*Oryzomonas rubra* Red88^T^	GCF_008369015.1		[27]
*Geomonas oryzae* S43^T a^	GCF_004117875.1		[28]
*Geomonas edaphica* Red53^T^	GCF_004917075.1		[28]
*Geomonas ferrireducens* S62^T^	GCF_004917065.1		[28]
*Geomonas terrae* Red111^T^	GCF_004791675.1		[28]
*Geomonas fuzhouensis* RG17^T^	GCF_020179575.1		[30]
*Geomonas agri* RG53^T^	GCF_020179605.1		[30]
*Geomonas* sp. Red32	JAKLOY010000000		This study
*Geomonas* sp. Red276	BLXW01000000		This study
*Geomonas oryzisoli* RG10^T^	GCF_018986915.1	Included in KEGG	[25]
*Geomonas subterranea* RG2^T^	GCF_019063845.1	Included in KEGG	[25]
*Geomonas nitrogeniifigens* RF4^T^	GCF_019063885.1	Included in KEGG	[25]

^a^ Type species of each genus; ICNP: International Code of Nomenclature of Prokaryotes

In addition to the presence of *nif* genes on the genomes, we confirmed the nitrogen-fixing activities of bacterial strains from these clades. In this study, we demonstrated that two type species within *Geobacteraceae*, namely *Geomonas oryzae* S43^T^ and *Oryzomonas japonica* Red96^T^ (Table 1) [27, 28], were able to grow on N_2_ as the sole nitrogen source (Fig. 1b). While the acetylene reduction activity of some strains within *Geobacter* and *Geomonas* has been previously tested [25, 29, 40], the ammonium-independent steady growth of *Geomonas* and *Oryzomonas* suggests that nitrogen fixation is energetically available. The present result, combined with the previously reported N_2_-dependent growth and acetylene reduction activity of *Anaeromyxobacter* and *Geobacter* strains [26, 40], indicate that *Anaeromyxobacteraceae* and *Geobacteraceae* are likely to be physiologically relevant to nitrogen fixation. We suspected that the use of their genomes as references would yield a better sensitivity in shotgun metagenomic analyses of deltaproteobacterial diazotrophs.

### Global distribution of diazotrophs in terrestrial environments

**Fig. 2:**
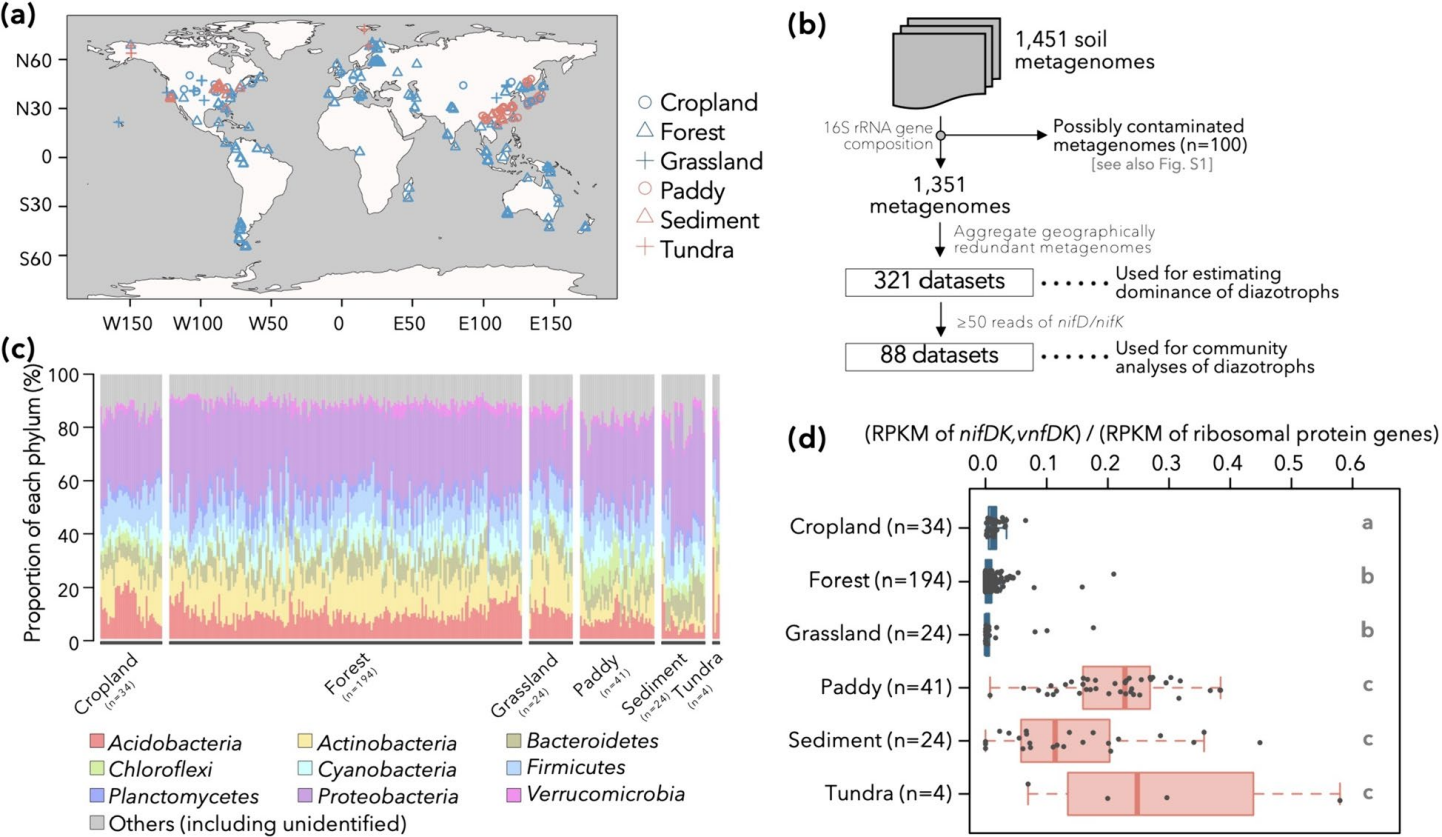
Overview of metagenomic datasets used in this study and distribution of diazotrophs therein. **a** The sampling locations for each metagenomic datasets used in this study. Six types of environments are differentiated by the shapes and colors of symbols. **b** Filtering procedure of metagenomic datasets. The filtering criteria, as well as the aggregation of geographically similar samples, are explained in the panel. **c** Phylum-level prokaryotic community structure of the 321 metagenomic datasets estimated by 16S rRNA gene sequences. **d** The dominance of nitrogen-fixing population in each environment. For each of the 321 metagenomic datasets, the ratio of reads per kilobase of reference sequence per million sample reads (RPKM) of nitrogenase genes to the RPKM of ribosomal protein genes is displayed. The letters on the right side of the box indicates the statistical significance in RPKM ratio between different environmental categories (*P* < 0.05, Brunner–Munzel test with Bonferroni’s correction). First, second, and third quantiles are indicated by solid lines. The whiskers, if any, denote 1.5*[interquartile range] from first or third quartile

To assess the global distribution of nitrogen-fixing populations, we collected 1,433 shotgun metagenomic datasets from public databases, namely NCBI SRA and MG-RAST [41, 42], coming from various environments including cropland soils, forest soils, grassland soils, paddy soils, sediments (including wetlands), and tundra soils (Fig. 2a and Table S1). Since metagenomes of Japanese soils (including volcanic soils) were poorly represented in public databases, we also collected 18 soil samples in Japan (Table S2) and sequenced their metagenomes. It should be noted that we only used metagenomic data in the present study, which indicate the quantity and diversity of potential diazotrophic microbes but do not serve as direct evidence of their diazotrophic activities.

Following preprocessing and curation of these datasets (i.e., merging of paired-end reads, quality filtering), we identified reads bearing nitrogenase genes (*nifDK* for molybdenum nitrogenase, *vnfDK* for vanadium nitrogenase, *anfDK* for iron-only nitrogenase [43]), 16S rRNA genes, or single-copy ribosomal protein genes conserved in most bacterial genomes [44] (Table S3). The phylogenetic compositions of 16S rRNA gene sequences indicated that 100 of these datasets could be contaminated by members of order *Lactobacillales* or plants’ plastids (amounting up to 80.5% of *Lactobacillales* or 71.6% of Chloroplast 16S rRNA gene reads: Fig. S1), and the remaining 1,351 were used for further analyses (Fig. 2b). Some of the 1,351 metagenomes were redundant (e.g., technical replicates), so we clustered metagenomes taken from environmental samples within 1 km of each other. This ended up in 321 samples (Fig. 2b), each considered to bear independent information. The 321 samples were overall dominated by well-known soil-dwelling bacterial clades such as phyla *Proteobacteria*, *Acidobacteria*, *Actinobacteria*, etc. (Fig. 2c) and showed no obvious hallmarks of abnormality or technical contamination.

From each of the 321 samples, we detected 110–5,714,345 reads (median: 8,050 reads) of ribosomal protein genes listed in Table S3. The number of nitrogenase gene reads were normalized by the number of ribosomal protein gene reads, taking into account the differences in gene lengths between orthologs (corrected by RPKM: see Materials and Methods). The relative abundances of nitrogenase gene reads were higher in paddy soils, sediments, and tundra soils (i.e., anaerobic environments) than those in aerobic environments, namely cropland, forest, and grassland soils (*P* < 0.05 in post-hoc pairwise Brunner–Munzel test with Bonferroni correction, Fig. 2d; Please note that only four data belonged to tundra). On average, nitrogenase genes were detected 17.6 times more frequently in the anaerobic samples than aerobic samples. This is consistent with both the well-established notion that biological nitrogen fixation is an anaerobic process and the oxygen-sensitive nature of nitrogenase [45].

Relative abundances of nitrogenase gene reads exhibited major variations among samples from aerobic environments (i.e., cropland, forest, and grassland), with some harboring low numbers, and others dominated by diazotrophs. Although the reason for such variation is not clear and require further experimental validation, here we list several hypotheses. First, several soil physicochemical properties, including total carbon, nitrogen, and available phosphorus contents, have been shown to affect the abundance of species which encode nitrogenase gene [16, 46]. It is also possible that cropland and grassland samples are affected by the roots of leguminous plants and nodule symbionts therein; alphaproteobacterial N_2_-fixing rhizobia such as *Bradyrhizobium*, *Azospirillum*, and *Mesorhizobium* were detected more frequently in legume crop soils than in non-legume crop soils [46, 47]. Some samples from aerobic environments may be locally anaerobic, and this might explain the variance in the relative abundance of diazotrophs. We also acknowledge the ambiguity in distinguishing forest or grassland soils from wetland sediments. For example, samples from Disney Wilderness Preserve (DWP), which are labeled as “area of pastureland or hayfields” in MG-RAST and presented the highest relative abundances of nitrogenase genes among “grassland” samples, may have originated from wetland-like environments, as the landscape of DWP bears patches of wetlands [48].

### Global diversity of diazotrophs in terrestrial environments

The taxonomic compositions of diazotrophic communities were further investigated for a more limited dataset of 88 samples, each of which comprised at least 50 sequences of *nifD/K* (Fig. 2b and Fig. S2). Please note that *nifD/K* serve as more accurate markers of diazotrophs compared with *nifH* (i.e., the conventional marker of diazotrophs [36]: see Materials and Methods for detail).

**Fig. 3:**
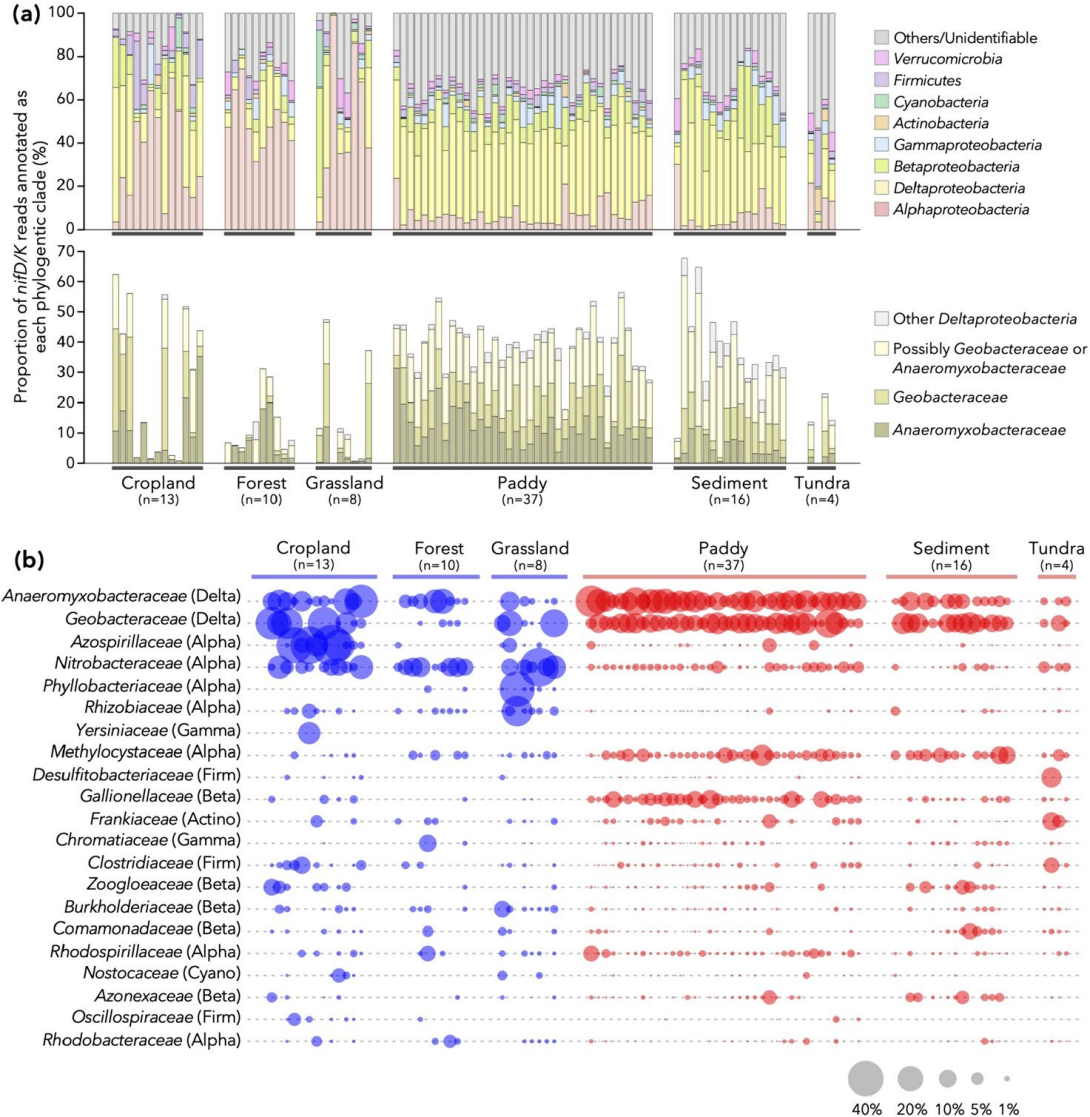
Phylogenetic compositions of nitrogenase genes in the metagenomic datasets with at least 50 reads of *nifD* and *nifK* (n = 88 in total). **a** Upper panel: phylum- and proteobacterial class-level composition. Lower panel: breakdown of deltaproteobacterial composition at the family level. The category “Possibly *Geobacteraceae* or *Anaeromyxobacteraceae*” comprises deltaproteobacterial reads that were unannotated at the family level but received higher-level annotations consistent with family *Geobacteraceae* or family *Anaeromyxobacteraceae*. **b** Family-level distribution of *nifD* and *nifK* reads. The correspondence with the phylum- and proteobacterial class-level taxonomy is noted in parentheses: Delta, *Deltaproteobacteria*; Alpha, *Alphaproteobacteria*; Gamma, *Gammaproteobacteria*; Beta, *Betaproteobacteria*; Firm, *Firmicutes*; Actino, *Actinobacteria*; Cyano, *Cyanobacteria*. The area size (not the radius) of each plot is proportional to the relative abundance of each family within each dataset

Reads encoding *nifD/K* from class *Deltaproteobacteria*, especially *Geobacteraceae* and *Anaeromyxobacteraceae*, were consistently dominant in anaerobic environments such as paddy soils and sediments, as well as in some of the aerobic samples (Fig. 3ab). While nitrogenase genes have a complicated history of horizontal gene transfer (HGT) that may hinder accurate taxonomic annotation [49, 50], Deltaproteobacterial NifD/K within Group I nitrogenase [49] are monophyletic (bootstrap value = 1.00, Fig. S3) and no clear hallmark of recent HGT [51]. Fortunately, a major part of deltaproteobacterial NifD/K within metagenomes belonged to this group (Fig. S4), and therefore it is unlikely that the dominance of *Deltaproteobacteria nifD/K* in this metagenomics is a byproduct of HGT. In addition, the proportion of deltaproteobacterial 16S rRNA genes (i.e., genes less prone to HGT) and frequency of deltaproteobacterial nitrogenase genes were significantly correlated (Fig. S5: Spearman’s *ρ* = 0.859 and *P* < 2.2×10^–16^ when tested using all samples; *ρ* = 0.738 and *P* < 2.2×10^–16^ when tested using only anaerobic samples). These results suggest that members of *Geobacteraceae* and *Anaeromyxobacteraceae* are one of the prominent drivers of nitrogen fixation in terrestrial ecosystems. While previous studies in wheat-soybean rotation croplands [16] and paddy soils [14, 52] are in line with our results, the metagenomic datasets analyzed here covering a wide range of environments provide a generalizable insight into the potential contributions of these clades to nitrogen fixation processes in the pedosphere.

Other major clades within nitrogen-fixing populations included *Alphaproteobacteria*, such as *Nitrobacteraceae* and *Rhizobiaceae* (Fig. 3ab), although some (e.g., *Bradyrhizobium* and *Rhizobium*) in these families are symbiotic diazotrophs and thus possibly incapable of independent nitrogen fixation outside their host plants. The community compositions were significantly different between the aerobic and anaerobic samples (permutational analysis of variance (PERMANOVA) of UniFrac distances, R^2^ = 0.114, *P* = 0.001), as further evidenced by a distinct grouping of the two types of samples in nonmetric multidimensional scaling (NMDS) analysis (Fig. S6).

Another characteristic of the diazotrophic communities of anaerobic environments (with the exception of tundra) is the high similarity between samples (Fig. 3). While all the paddy soil and sediment samples are dominated by *Deltaproteobacteria* and present overall low beta-diversity levels, environments such as cropland, forest, and grassland are dominated by more diverse clades of diazotrophs showing high beta-diversity levels. One possible explanation is the heterogeneity among aerobic samples: some of the cropland, forest, and grassland samples may be associated with leguminous vegetations (i.e., affected by nodule-associated bacteria) [18, 46] or originated from soil physicochemical properties/conditions [16], but they are not explicitly considered in this study. Another explanation for this divergence in community structures is ecological drift [53, 54]. Diazotrophs have smaller population sizes in aerobic samples (Fig. 2d); thus, their communities are expected to be more sensitive to ecological drift, resulting in increased beta-diversities between communities as previously shown [55].

Although we used a limited dataset of 88 samples bearing at least 50 *nifDK* sequences for the taxonomic composition analysis, the bias introduced by this manipulation is unlikely to be critical. First, the selected samples do not necessarily present high relative abundances of diazotrophs, since the number of total reads greatly varies between samples (Fig. S2ab). Second, the abundance ratio of nitrogenase genes to ribosomal protein genes explain only 5.0% the phylogenetic diversity of *nifDK* (R^2^ = 0.050, *P* = 0.036) within aerobic samples (Fig. S2c).

As a side note, we also analyzed the abundance and phylogeny of *nifH*, a conventional marker for nitrogen-fixing populations. Because the lengths of *nifH*, *nifD*, and *nifK* genes are approximately 3:5:5 (Table S3), the number of these genes should also be around 3:5:5 in each metagenome. However, some samples harbored disproportionally higher number of *nifH* compared with *nifD/K*: nine of the metagenomic samples included 1.5 times or higher number of *nifH* reads than can be expected from the number of *nifD/K* reads (blue points in Fig. S7a). This implies that some soil samples bear significant amount of pseudo-*nifH* genes [36], which are encoded on prokaryotic genomes lacking other essential components of nitrogenase genes (e.g., *nifD* and *nifK*). We suspect that *nifD/K*, rather than *nifH*, serve as a reliable marker gene for nitrogen-fixing populations (especially in shotgun metagenomic studies). Regarding 79 samples with lower amounts of pseudo-*nifH*, deltaproteobacterial *nifH* were dominant (Fig. S7b) in congruence with the results of *nifDK* analyses (Fig. 3a).

### An approximate estimation of the global dominance of deltaproteobacterial diazotrophs

Analyses of the global metagenomic dataset indicated that anaerobic environments harbor high abundances of diazotrophic prokaryotes and that *Anaeromyxobacteraceae* and *Geobacteraceae* are the dominant diazotrophs in these environments. Wetlands (possibly including waterlogged paddy soils) represent between 5.2% [56] and 8% [57] of all lands, with microbial biomass carbon therein amounting to 10.3% of the microbial biomass in all lands [calculated from the data presented in [56]]. Thus, although wetland is a limited area of land, given that the relative abundance of nitrogen fixers was 17.6 times higher in anaerobic microbial communities than in aerobic ones (Fig. 2d), wetland could be a large reservoir of nitrogen fixers on terrestrial environments.

### Biases behind amplicon sequencing of nitrogenase genes

The prevalence of diazotrophic *Geobacteraceae* has actually been reported in some of PCR amplicon sequencing analyses of *nif* genes [15, 16, 58], but the dominance of *Anaeromyxobacteraceae* has been overlooked in such PCR-based analyses. We suspected that this discrepancy is due to PCR biases behind amplicon sequencing. It is commonly accepted that results of amplicon sequencing are dependent on a series of PCR conditions such as primer sets and DNA polymerases [37, 59]. The GC contents of templates, as well as primer mismatches, can also affect the amplification efficiency and therefore cause biases [34, 35, 37]. Notably, *nif* genes of *Anaeromyxobacteraceae* have higher GC contents (65.6–69.7%) than those of the other bacteria (Fig. S8).

To elucidate the PCR biases of *nif* genes, we performed amplicon sequencing of nitrogenase genes in six soil DNA samples and compared the results directly with shotgun sequencing of the same samples. We prepared amplicon libraries under ten PCR conditions with different primer sets and DNA polymerases (Tables S4 and S5). Please note that we here targeted *nifH*, rather than *nifDK*, for the sake of consistency with conventional amplicon sequencing methods. Primer mismatches will not be extensively discussed here, because *nifH* of *Anaeromyxobacteraceae* and other clades present similar identities to the primers (Fig. S9).

As expected, we found that the phylogenetic compositions of *nifH* amplicons were dependent on type of DNA polymerases and primer sets (Fig. S10a–f), and the discrepancy was particularly remarkable in the proportion of *Anaeromyxobacteraceae nifH*. *Anaeromyxobacteraceae nifH* were consistently more highly represented in KOD One libraries than in DreamTaq libraries (Fig. S10g). In addition, their proportion in shotgun metagenomic sequences were comparable to those in KOD One libraries, although dependent on sample identities and primer sets (Fig. S10h). These suggest that DreamTaq failed to amplify *Anaeromyxobacteraceae nifH*. What we focus on here is the high GC contents of *nif* genes in *Anaeromyxobacteraceae* (Fig. S8). According to the manufacturers’ reports (https://lifescience.toyobo.co.jp/user_data/pdf/products/manual/KMM-101_201.pdf [in Japanese; accessed Jan 5, 2024]), KOD One is robust to amplify GC-rich templates, while DreamTaq shows a low performance (https://www.thermofisher.com/order/catalog/product/EP1701?SID=srch-srp-EP1701 [accessed Jan 5, 2024]), which aligns with the present results. We speculate that GC richness of *Anaeromyxobacteraceae nifH* may be one reason why they have been poorly represented in amplicon surveys.

We also argue that these results represent the major biases behind amplicon sequencing. Provided that *nifH* gene compositions were largely dependent on the type of DNA polymerases and primer sets, comparing the results obtained from multiple studies might be difficult. In this respect, meta-analysis of shotgun metagenomic data should be a straightforward, solid, and less biased approach (as has been discussed in Kim et al. [60]).

Even in shotgun metagenomic sequencing, it should be noted that the abundance of diazotrophic *Anaeromyxobacteraceae* may be underestimated. First, library preparation for shotgun sequencing often involves PCR (typically 8–12 cycles), which may fail to amplify GC-rich nucleotide fragments [61, 62]. Second, Illumina sequencing technology is known to be biased against sequencing GC-rich nucleotide fragments even in shotgun sequencing [63]. Considering these biases, the proportion of *Anaeromyxobacteraceae* in the soil, the nitrogenase genes of which are GC-rich (66.9%–69.0%, 65.6%–67.0%, and 66.9%–69.7% for *nifH*, *nifD*, and *nifK*, respectively, Fig. S8), might be even higher than estimated in this study. The former issue may be addressed using PCR-free library preparation protocols [62]. Long-read sequencers (i.e., PacBio and Nanopore) are less prone to GC bias [63] and potentially rectify the latter issue, although their current yield is orders of magnitude smaller than those of short-read sequencers such as Illumina HiSeq and NovaSeq, and thus currently not a good fit for the characterization of samples as heterogenous and rich in diversity as soil metagenomes.

### Benefits of expanding culture collection

**Fig. 4:**
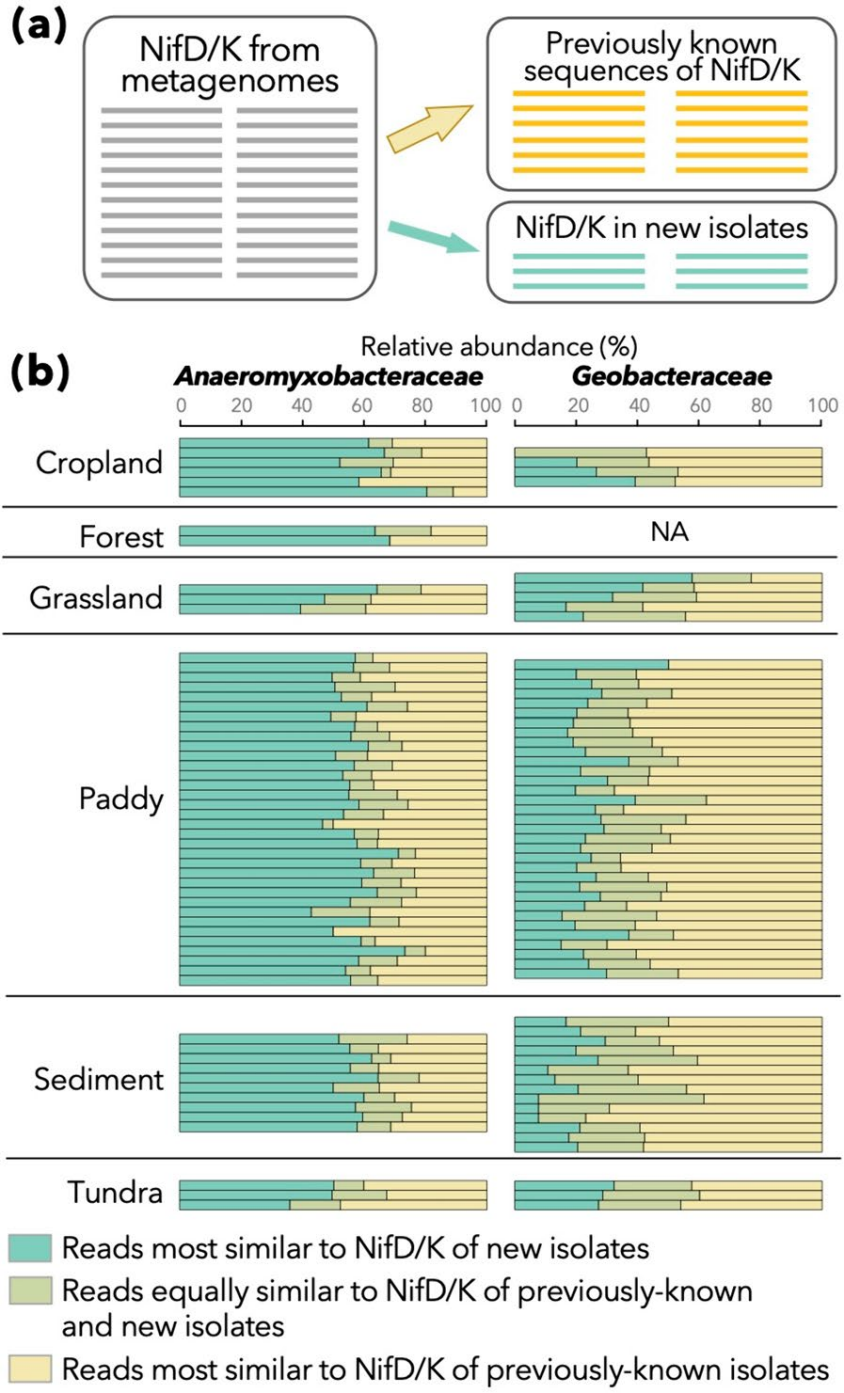
Contribution of nitrogenase gene sequences from newly isolated strains in the bioinformatic analyses of metagenomes. **a** A schematic of the analysis. NifD/K sequences annotated as *Anaeromyxobacteraceae* or *Geobacteraceae* in metagenomes were mapped onto already known sequences of NifD/K (right-upper) and those in our new isolates (right-bottom). Only the top hit for each query sequence (i.e., one from metagenomes) was considered. **b** Relative abundance of metagenome-derived NifD/K sequences that were most similar to already known sequences (yellow) and those from our new isolates (green), as well as those equally similar to the nitrogenase genes of already known genomes and our new isolates (dim green), are summarized. Only datasets with 10 or more sequences of NifD/K for each family are displayed

Previous and current efforts to enrich culture collections [23, 24, 26–29, 31] have substantially expanded the available repertoire of *Anaeromyxobacteraceae* and *Geobacteraceae* strains. In fact, an average of 56.9% and 23.9% of NifD/K sequences derived from *Anaeromyxobacteraceae* and *Geobacteraceae* members, respectively, displayed higher similarity to our novel strains than to any other nitrogenase sequence in KEGG from these families (Fig. 4).

Interestingly, this trend was consistent among a wide variety of environments including aerobic and anaerobic environments, although the majority of the novel strains were isolated from paddy soils or sediments under anaerobic conditions. Based on the present and previous findings, paddy soils and sediments appear to be promising environments for isolating free-living diazotrophs, representing diverse terrestrial environments including aerobic environments such as cropland, forests and grassland.

## Conclusions and outlook

Contrary to the conventional view, our large-scale comparative metagenomics analyses revealed the global distribution and substantial abundance of *Anaeromyxobacteraceae* and *Geobacteraceae* in terrestrial diazotrophic microbiome, highlighting the potential importance of *Deltaproteobacteria* members (phyla *Myxococcota* and *Desulfobacterota*) in terrestrial, especially anaerobic, ecosystems. Although *Anaeromyxobacteraceae* and *Geobacteraceae* have been well known as iron- and other metals- reducing bacteria in soil environments, this study is the first to report that they are the most dominant group of terrestrial diazotrophic microbiome on a global scale.

Moreover, nitrogen-fixing bacteria have long been considered useful microorganisms for improving soil nitrogen fertility, and methods to promote their activity have been developed for sustainable agriculture [64]. For example, in paddy soils, recent studies showed that application of iron-bearing materials could enhance the nitrogen-fixing activities of indigenous iron-reducing bacteria within the families *Anaeromyxobacteraceae* and *Geobacteraceae* and maintain rice yields under reduced nitrogen-fertilizer application [65, 66]. Given the ubiquity of iron-reducing diazotrophs (Fig. 3), this strategy may be effective in a variety of other crop fields. More generally, careful and precise updates of our understanding of functional microorganisms in soil environments should advance such attempts towards sustainable agriculture.

It should be noted that the pivotal thing for microbiome discovery is to improve the accuracy of metagenomics, i.e., to expand the available genomic information of microorganisms. In this study, thousands of obtained nitrogenase sequences exhibited high proximity to our newly isolated strains. Our results warrant further efforts to improve culture collections, which would fill the knowledge gaps in the diversity and ecology of diazotrophs. Especially in soil environments, the enormity of uncultured but predominant clades of prokaryotes, as represented by members of *Acidobacteria* and *Verrucomicrobia* [67–69], is widely recognized. To advance our knowledge of the terrestrial diazotrophic microbiome, strategies for their cultivation and isolation should be also updated, for example, by using single-cell sorting.

There is no doubt that *Anaeromyxobacteraceae* and *Geobacteraceae* are important diazotrophic members in soils that should not be underestimated or undervalued as they have been. However, unfortunately, it is impossible to estimate how much *Anaeromyxobacteraceae* and *Geobacteraceae* actually contribute to nitrogen fixation in soil environments based on the results of this study alone, since the contribution cannot be directly inferred from the detected amount of genes. The insights into the contribution of each diazotrophic taxon to terrestrial nitrogen fixation will be foreseeable, for instance, through stable isotope probing (SIP) with ^15^N_2_ under more natural conditions, using various soil samples. Although preliminary, a recent study based on ^15^N-DNA-SIP analysis revealed a high contribution of *Anaeromyxobacteraceae* and *Geobacteraceae* for nitrogen fixation in a paddy soil [70], supporting our conclusion.

## Materials and methods

### Isolation and genomic sequencing of new soil strains

The *Geomonas* strains Red32 and Red276 were isolated from paddy soil (Joetsu, Niigata, Japan) and pond sediment (Myoko, Niigata, Japan) following the slurry incubation method used to isolate new members of *Geobacteraceae* [27, 28]. The soils collected from the paddy field in Nagaoka, Niigata, Japan were air-dried, placed in a 15-mL serum bottle and suspended in distilled water (soil:water, 2:3, *w*/*v*). After autoclaving at 120°C for 20 min, 0.1 g of undried soil was added to the bottle as a microbial inoculum with and without vitamin solution for strains Red276 and Red32, respectively [26]. Then, we sealed the bottles with butyl rubber stoppers and aluminum caps, replaced headspace gas with with N_2_/CO_2_ (80:20, *v*/*v*), and incubated them at 30°C for 2 weeks without shaking. Afterward, 200 μL of incubated soil slurry was transferred to a new bottle of autoclaved soil slurry and incubated at 30 °C for 2 weeks. After repeating this step once (for strain Red276) or twice (for strain Red32), the incubated soil slurry was streaked on 1.5% agar plates of the R2A broth “DAIGO” (Nihon Pharmaceutical, Tokyo, Japan) supplemented with 5 mM disodium fumarate. The plates were incubated at 30°C for 10 days under anaerobic conditions using the AnaeroPack system (Mitsubishi Gas Chemical, Tokyo, Japan). Red-colored colonies, a typical hallmark of *Geobacteraceae* strains [23, 27, 28, 71, 72], were purified by a single-colony isolation using the same medium plates. Genomic DNA was extracted from the two isolated strains using a DNeasy Blood and Tissue Kit (Qiagen, Hilden, Germany) and sequenced using an Illumina HiSeq sequencer (Illumina, CA, USA) for 2×150 paired-end configuration. The resulting sequences were assembled using Velvet v1.2.10 [73] as previously described [27, 28].

### Diazotrophic activity assay

Following a 5-day culture in nitrogen-free modified freshwater medium (MFM) as previously described [27, 28], the cells of *Geomonas oryzae* S43^T^ and *Oryzomonas japonica* Red96^T^ were transferred to serum bottles containing 20 mL of nitrogen-free MFM [28] and headspace gas was replaced with N_2_ gas. No contamination of ammonia in used N_2_ gas was confirmed by no growth of non-diazotrophic *Anaeromyxobacter* strain, *A. dehalogenans* 2CP-1^T^ [26]. Bacterial growth was monitored by measuring the suspension absorbance using a spectrophotometer (UV-1900 UV-visible spectrophotometer, Shimadzu, Kyoto, Japan) at a wavelength of 600 nm. The experiments were performed in triplicate.

### Preparation of custom database

We used KEGG database (as of August 31, 2022) for functional gene annotations [74]. To increase the sensitivity for genes from *Anaeromyxobacteraceae* and *Geobacteraceae*, we customized KEGG database by adding genomes belonging to these families obtained using slurry incubation methods (Table 1). Genomes already included in KEGG were not added. We predicted their coding sequences (CDS) using Prodigal [75] with default parameters, annotated them using KofamScan version 1.3.0 and KOfam version 2022-08-01 with default parameters [76], and concatenated the CDS with KEGG database (including those received no K number). The phylogenies of NifD and NifK within Group I [49] were determined using MAFFT v7.505 (with “--auto” option) and FastTree 2.1.11 [77, 78] with a bootstrap test of 100 iterations (otherwise default parameter settings). For the bootstrapping tests, we also used “CompareToBootstrap.pl” script (http://www.microbesonline.org/fasttree/treecmp.html, accessed April 18, 2023) to merge the resampled trees.

### Phylogenetic analysis of bacterial genomes harboring *nif* genes

From the aforementioned custom database, we screened genomes harboring a set of *nif* core genes, namely *nifH* (K02588 in KEGG), *nifD* (K02586), and *nifK* (K02591). Archaeal genomes were excluded from the analysis. The universal single-copy gene sequences were identified from each genome, translated amino acid sequences, and mapped onto multiple sequence alignment (MSA) of GTDB R207 using GTDB-Tk v2.1.0 [44, 75, 79, 80]. Here “identify” and “align” commands were used with default parameter settings. The MSA was fed into FastTree (default parameters) and a phylogenetic tree was constructed. The tree was manually rerooted using *Cyanobacteria* as the outgroup [81] and visualized on the iTOL server [82].

### GC content of 16S rRNA genes and nitrogenase genes among bacterial genomes

Ribosomal RNA genes were identified from each of the bacterial genomes in the custom database explained above using barrnap version 0.9 (https://github.com/tseemann/barrnap; accessed April 18, 2023). Only 16S rRNA sequences with 1000 bases or longer were picked. For each genome with at least one valid 16S rRNA gene sequence and all of the identified *nifH*, *nifD* and *nifK* (identified as previously described), the GC contents of 16S rRNA genes, *nifH*, *nifD*, and *nifK* were calculated. When a genome had multiple copies of each gene, GC content was calculated for the concatenated sequence of these copies. Any ambiguous base was excluded from the calculation of GC content.

### Soil collection and shotgun metagenomic sequencing

We collected 18 surface soil samples from various agricultural fields in Japan at an approximate depth of 0–5 or 0–10 cm (Table S1). Following the removal of plant residues and additional water from the surface, the soil samples were stored at −80 °C or −30 °C until further use for DNA extraction. Soil DNA was extracted from 0.5 g (wet weight) of each soil sample using the ISOIL for Beads Beating Kit (Nippon Gene, Tokyo, Japan) according to the manufacturer’s instruction with the following modifications: prior to the beads beating step, 0.02 g skim milk was added to the lysis buffer to improve the extraction efficiency [83] and post-elution purification using RNase A (Takara, Shiga, Japan) and DNA Clean & Concentrator (Zymo Research) according to the manufacturer’s introduction. Purified DNA was quantified using Qubit 2.0 Fluorometer (Invitrogen, Carlsbad, CA, USA) with Qubit dsDNA HS Assay Kits (Invitrogen). The construction of DNA libraries, shotgun sequencing on an Illumina MiSeq sequencer, and merging of paired-end sequences were performed as described previously [14]. Regarding the other 6 soil samples, DNA was extracted from 0.25 g of each soil using DNesay PowerSoil Pro Kits (QIAGEN, Hulsterweg, Netherland) following the manufacturer’s instruction. Shotgun sequencing library was prepared using MGIEasy FS DNA Library Prep (MGI Tech, Guangdong, China), where the duration of fragmentation reaction was customized to four minutes and library amplification was performed for eight cycles. MGIEasy Circularization Kit and DNBSEQ-G400RS High-throughput Sequencing Kit Set were used to construct DNBs, which were sequenced on DNBSEQ-G400 (MGI Tech) under 2x200 bp paired-end mode. Soil pH(H_2_O) and electrical conductivity were measured in a suspension sample with soil-water ratio of 1:5 (w/w). Soil total carbon and nitrogen contents were determined using dry combustion method. Crop types and chemical properties of Japanese soils used in this study were summarized in Table S2.

### Collection of publicly available metagenomic data and their quality assessment

We further collected reusable datasets of bulk soil metagenomes on INSDC [84] and MG-RAST [85] that met the following criteria: (i) derived from outdoor samples exempted from post-sampling treatments that can affect the microbial community structure; (ii) sequenced on Illumina MiSeq, HiSeq, MiniSeq, NextSeq or NovaSeq (i.e., state-of-the-art, highly accurate sequencers); and (iii) reported in the peer-reviewed literature (with the exception of data obtained by the National Ecological Observatory Network). Moreover, the datasets from rhizosphere soils were not used in this study because they are extensively and dynamically affected by the plant roots [86] and not representative of the soil microbial communities. In total, we collected 1451 datasets as listed in Table S1 [47, 87–121]. The latitude and longitude of each sampling site were obtained from public databases [INSDC BioSamples database [122] and MG-RAST] and verified with the descriptions in each publication. The INSDC data were directly obtained from DDBJ server, whereas those on MG-RAST were fetched using MG-RAST API (with the option “file=050.1”).

The collected metagenomic data underwent extensive curations, followed by homology searches to detect nitrogenase genes, ribosomal protein genes, and 16S rRNA genes. Detailed procedures are provided in the supplementary information. After a series of data curation, we decided to use 1,333 metagenomes from public databases and newly sequenced 18 metagenomes for downstream analyses. Some of the metagenomes were geographically redundant, so we merged metagenomes from samples taken within < 1 km and treated them as one sample. The distances between sampling locations were calculated based on the latitude and longitude of each sample using the geodesic module in GeoPy (https://geopy.readthedocs.io/en/stable/#; accessed Jan 5, 2024).

### Gene annotations of metagenomic reads

To determine the nitrogen-fixing populations within each metagenomic dataset, the filtered sequences were subjected to homology search against the custom database explained above (i.e., KEGG database supplemented with *Anaeromyxobacteraceae* and *Geobacteraceae* genomes), followed by the taxonomic annotation of nitrogenase gene reads. In short, we determined the relative abundance of nitrogenase-harboring prokaryotes and their taxonomic composition for each sample.

Although the details are explained in the supplemental text, here we note three key strategies. First, we used *nifD*, *nifK*, *vnfD*, *vnfK*, *anfD*, and *anfK* as the marker genes. *nifH* was not used for this purpose because the partial primary structure of NifH can be confused with those of other proteins irrelevant to nitrogen fixation [36]. Second, we normalized the number of reads by those of single-copy prokaryotic ribosomal protein genes [81], rather than by the total number of metagenomic reads that may be affected by plant- and animal-derived sequences. Third, we used phylogenetic placement, rather than a simple homology search, for taxonomic annotation of *nif* gene reads. The reliability of each taxonomic annotation was calculated based on the likelihood of phylogenetic relationships between metagenomic reads and reference sequences, and therefore we were able to abandon uncertain annotations. For example, short fragmented reads may bear little phylogenetic signals, and annotations of such reads were to be unreliable and discarded.

### Beta-diversity analyses

Beta-diversity between any pair of diazotrophic communities was calculated using the average of UniFrac distances for NifD and NifK, which were determined based on the results of phylogenetic placement. We used NMDS with two dimensions to summarize overall beta-diversity between communities. We also performed PERMANOVA with 999 times permutation to test the null hypothesis that community structures of diazotrophs are similar between aerobic and anaerobic environments.

### Homology analyses between NifD/K of metagenomes and isolate genomes

We further mapped the NifD/K sequences annotated as family *Anaeromyxobacteraceae* or *Geobacteraceae* in metagenomic reads to NifD/K sequences from that family in our custom database using the Needleman–Wunsch algorithm implemented in USEARCH v11.0.667 [123]. We obtained the sequence similarity between each read and its nearest sequence in the database. We counted the number of reads for which the nearest sequence is from the genomes of bacterial isolates obtained via the slurry incubation method (Table 1).

### Amplicon sequencing of nitrogenase genes using popular primer sets

We compared the results of shotgun metagenomic sequencing and amplicon sequencing of nitrogenase genes using six of the Japanese soil samples. Using the four pairs of universal primers (Table S4) and three DNA polymerases that differ in performance (DreamTaq DNA Polymerase [ThermoFisher Scientific], Ex Taq Hot Start Version [Takara], and KOD One [TOYOBO]), and we amplified *nifH* genes contained in each soil metagenome. Here we performed two-step tailed PCR to construct Illumina library, consisting of the first PCR to amplify *nifH* genes and the second PCR to attach index sequences to the amplicons. The amplicon of first PCR were cleaned up using AMPure XP (Beckman Coulter, Brea, CA, USA) before subjected to the second round of PCR. Detailed PCR conditions are summarized in Tables S4 and S5. The final PCR products were electrophoresed on agarose gels, purified using Wizard® SV Gel and PCR Clean-Up System (Promega, Madison, WI, USA), and sequenced on Illumina iSeq in a paired-end mode (151 bp × 2). For each combination of soil samples, primer pairs, and DNA polymerases, we amplified *nifH* genes and sequenced them in triplicates. The obtained reads underwent error correction using DADA2 [124], and the amplicon sequence variants (ASVs) were further filtered to eliminate chimeras and non-specific amplicons. The filtered ASVs were taxonomically annotated using phylogenetic placement. Details are explained in supplemental method.

### Estimation of mismatches between *nif*-harboring genomes and *nifH* universal primers

To determine the mismatches between prokaroytic nitrogenase genes and their universal primers, we mapped sequences of seven primers (PolF, PolR, nifH-F, nifH-R, Ueda19F, Ueda407R, and univ463r: Table S4) onto 12 prokaryotic *nifH* sequences (*Anaeromyxobacte*r sp. Fw109-5, *Anaeromyxobacter* sp. K, *A. diazotrophicus* Red267^T^, *A. oryzae* Red232^T^, *A. paludicola* Red630^T^, *Azospirillum brasilense* Sp7^T^, *Azotobacter vinelandii* DJ, *Bradyrhizobium diazoefficiens* USDA110^T^, *Clostridium acetobutylicum* ATCC824^T^, *Frankia casuarinae* CcI3^T^, *Geomonas oryzae* S43^T^, and *Oryzomonas japonica* Red96^T^). We referred to annotations on KEGG or NCBI RefSeq to collect *nifH* sequences. In cases where one genome owned multiple copies of *nifH*, we selected one copy that was accompanied by *nifDK* in their neighborhood [36]. The collected *nifH* genes were aligned using MAFFT v7.505 (with “--auto” option), and then the primer sequences were manually aligned onto the MSA.

Throughout the study, we used SeqKit v0.16.1/v2.2.0 [125] and R 4.0.5/4.1.1 [126], including the package “vegan” [127], to handle fastq and fasta files and to perform statistical tests, respectively.

### Supplementary Information

**Supplementary Material 1.**

**Supplementary Material 2.**

**Supplementary Material 3.**

**Supplementary Material 4.**

## Data Availability

Genomic sequences obtained in this study have been deposited in GenBank. Shotgun metagenomic and amplicon sequences have been deposited in DDBJ DRA. See Tables 1, S1, and S6 for accession numbers.
