# Supplementary material for: Global soil metagenomics reveals distribution and predominance of Deltaproteobacteria in nitrogen-fixing microbiome
Source: Microbiome. 2024 May 24;12:95. doi: 10.1186/s40168-024-01812-1 (PMC11127431; doi:10.1186/s40168-024-01812-1)
Supplement: Supplementary file 2 — Supplementary Material 1. [file 40168_2024_1812_MOESM1_ESM.pdf]

Supplementary information

Global soil metagenomics reveals distribution and predominance of *Deltaproteobacteria* in nitrogen-fixing microbiome

Yoko Masuda [a,b*], Kazumori Mise [c*], Zhenxing Xu [a], Zhengcheng Zhang [a], Yutaka Shiratori [d], Keishi Senoo [a,b], and Hideomi Itoh [c]
(*: Equally contributed)

[a] Department of Applied Biological Chemistry, Graduate School of Agricultural and Life Sciences, The University of Tokyo, 1-1-1 Yayoi, Bunkyo-ku, Tokyo 113-8657, Japan
[b] Collaborative Research Institute for Innovative Microbiology, The University of Tokyo, 1-1-1 Yayoi, Bunkyo-ku, Tokyo 113-8657, Japan
[c] National Institute of Advanced Industrial Science and Technology (AIST) Hokkaido, 2-17-2-1 Tsukisamu-higashi, Toyohira, Sapporo, Hokkaido 062-8517, Japan
[d] Niigata Agricultural Research Institute, 857 Nagakura-machi, Nagaoka, Niigata 940-0826, Japan

Corresponding authors:
Yoko Masuda (yokomasuda@g.ecc.u-tokyo.ac.jp)
Kazumori Mise (mise-33@aist.go.jp)
Hideomi Itoh (hideomi-itou@aist.go.jp)

SUPPLEMENTARY METHODS

1. Quality control of metagenomic datasets

As described in the main text, we collected 1451 metagenomic datasets from public databases or by ourselves. The collected metagenomic sequences were formatted and filtered as follows.

The paired-end sequences were merged using the "fastq_mergepairs" command in USEARCH v11.0.667 (Edgar, 2010) with the options "-fastq_maxdiffs 5 -fastq_minovlen 15". As a rule, merging of paired-end reads is strongly influenced by the length of the Illumina library, which differs widely between research projects. In particular, read pairs from long libraries (e.g., those designed for metagenomic assembly) are rarely merged. Therefore, we used Read 1 sequences that failed to be merged in addition to successfully merged sequences and single-end sequences. From each of the merged and unmerged sequences, we picked a partial sequence with an expected number of errors of fewer than 0.5 bases. From these partial sequences, those shorter than 100 bases were discarded; therefore, the expected error rate of each sequence was approximately 0.5% (i.e., 0.5 bases per 100 bases) or lower.

To determine the prokaryotic community structure of each dataset, we identified 16S rRNA gene sequences from these filtered sequences, using SortMeRNA v2.1 (Kopylova et al., 2012) with SILVA v138.1_SSURef_NR99 (Quast et al., 2012) as the reference database. Retrieved 16S rRNA gene sequences were further filtered by BLASTn search against SILVA at the maximum e-value threshold of 1e–10 and a minimum query coverage of 50%. Those passing this filter were taxonomically annotated using SINTAX (Edgar, 2016) implemented in USEARCH v11.0.667 with a minimum confidence threshold of 0.5. Here again, SILVA was used as the reference database, with the following modifications to maximize the taxonomic annotation accuracy: SILVA taxonomy was replaced with NCBI taxonomy (referred to taxmap_embl-ebi_ena_ssu_ref_nr99_138.1), and sequences without a valid taxonomic assignment at phylum, class, or order level (typically metagenome-derived sequences) were eliminated. However, organelle sequences (i.e., sequences annotated as mitochondrial and chloroplast according to SILVA taxonomy) were exceptionally retained.

We excluded 100 metagenomic datasets, with a proportion of *Lactobacillales* or Chloroplast 20% or higher (Fig. S1), from downstream analyses because they were considered possibly contaminated. The remaining 1333 metagenomes from public databases and newly sequenced 18 metagenomes, including those from samples taken within < 1 km, were merged and treated as one sample. The distances between samples were calculated based on the latitude and longitude of each sample using the geodesic module in GeoPy (https://geopy.readthedocs.io/en/stable/#; accessed Jan 5, 2024).

2. Bioinformatic analyses of metagenomes

Here we describe the bioinformatic details for metagenomic annotations. First, we identified nitrogenase genes and ribosomal protein genes; second, we calculated the relative abundance of prokaryotes harboring nitrogenase genes within each metagenome; and third, we put taxonomic annotations on nitrogenase genes.

2.1. Identification of nitrogenase genes and ribosomal protein genes

To identify the reads bearing nitrogenase genes or ribosomal protein genes, we used a two-step homology search (Yu and Zhang, 2013). First, we mapped the query sequences against nitrogenase amino acid sequences [K02586 (NifD), K02591 (NifK), K22896 (VnfD), and K22897 (VnfK)] using the blastx command implemented in DIAMOND v2.0.9.147 (Buchfink et al., 2021) with the options "--sensitive -e 10e-5". NifH was not used in this analysis because of its primary structure, which can be confused with those of other proteins irrelevant to nitrogen fixation (Mise et al., 2021). Query sequences aligned with nitrogenase sequences, which may be regarded as the candidate *nif* genes, were subjected to a second screening. Specifically, CDSs were inferred from these candidates using Prodigal (metagenomic mode) and mapped against the whole KEGG database to determine whether they were *nif* genes. Once again, the blastp command implemented in DIAMOND was used with the options "--sensitive -evalue 10e-5". To avoid possible errors caused by the heuristic nature of DIAMOND searches, we obtained up to 200 hits for each query and determined one top hit among them according to the bit scores. In addition, we abandoned amino acid sequences shorter than 50 aa. Then, a query with a top hit against a K02586 [K02591, K22896, or K22897] sequence was regarded as a *nifD* [*nifK*, *vnfD*, or *vnfK*, respectively] sequence. Note that the top hit was used only to differentiate *nif* genes from other genes, rather than the taxonomic assignment of each query.

2.2. Calculating the relative abundance of prokaryotes harboring nitrogenase genes within community

To normalize the abundance of the nitrogen-fixing population by the size of the prokaryotic population, we counted the number of reads bearing prokaryotic ribosomal protein genes, single-copy markers conserved among most prokaryotes known to date (Hug et al., 2016). The genes used for this purpose are listed in Table S3, and the reads bearing these genes were identified in the same way as the *nif* genes. Because the length of each marker gene was different (Table S3), we calculated the reads per kilobase of reference sequence per million sample reads (RPKM) for each ribosomal protein gene and used their median as the representative RPKM of the dataset. The length of reference sequence was substituted with the triple of the average length of amino acid sequences in KEGG. We also calculated the RPKMs

of four *nif* genes (*nifD*, *nifK*, *vnfD*, and *vnfK*) to compare them with those of ribosomal proteins. The sum of the four RPKMs was regarded as the "total" RPKM of the *nif* genes. Then we calculated the ratio of the "total" RPKM of the *nif* genes and the median RPKM of the ribosomal protein genes, which represent the population of diazotrophs and total prokaryotes, respectively. This ratio roughly represents double the proportion of diazotrophs to all prokaryotes, as most diazotrophs carry the gene pair "*nifD* and *nifK*" or "*vnfD* and *vnfK*." For any pair of the environmental categories, a post hoc Brunner–Munzel test was performed to test the null hypothesis that RPKM ratios are the same between the two categories.

2.3. Taxonomic assignments and classifications of metagenomic reads encoding *nif* genes

The taxonomic annotation of protein-coding genes is a cumbersome and error-prone process because of the rapid evolution of sequences. To make most of the fragmented metagenomic sequences while averting annotation errors, we employed phylogenetic placement, which is implemented in pplacer (Matsen et al., 2010), rather than a simple homology search. The effectiveness of introducing phylogenetic information into taxonomic assignment was previously demonstrated (Kapili and Dekas, 2021). Briefly, an MSA of reference NifD (or NifK) sequences as well as a backbone phylogenetic tree of NifD (or NifK) was constructed. The metagenomic sequences of NifD (or NifK) were mapped onto the backbone tree and were taxonomically annotated. First, NifD (or NifK) sequences in KEGG [under the K numbers K02586 (or K02591)] and our isolates (listed in Table 1) were fed into MAFFT v7.475 (with the option "--auto") (Katoh, 2002). In this analysis, the identical sequences of NifD or NifK were dereplicated because pplacer's implementation is incompatible with the zero branch length. None of the identical sequences were derived from different families with only one exception; therefore, this manipulation would not compromise the rigor of taxonomic annotation. From the MSA, an approximate maximum likelihood tree was constructed using FastTree (Price et al., 2009). The tree was automatically rerooted using taxtastic v0.9.2 (distributed along with pplacer) and subsequently used as a backbone phylogenetic tree for phylogenetic placement.

Each NifD/K-like amino acid sequence from the metagenomes was mapped onto the MSA using MAFFT with the option "--add". That sequence was placed onto the backbone tree using pplacer v1.1.alpha19-0-g807f6f3 (with the option "--mrca-class"), and its phylogenetic taxonomy was determined using guppy v1.1.alpha13-0-g1ec7786 (https://matsen.github.io/pplacer/generated_rst/guppy.html) (with the option "classify --mrca-class"). pplacer and guppy report several or dozens of solutions (i.e., taxonomic annotations) with likelihood values, which enabled us to assess the reliability of each annotation. We used family-, order-, class-, and phylum-level annotations with a likelihood value of 0.5 or higher (the threshold was precisely set at 0.49999 to consider the effect of floating-point errors).

Theoretically, two different solutions could satisfy this criterion (with the likelihood values of 0.5 each) for one query, but practically, such a situation did not occur. When none of the annotations satisfied this criterion, the sequence in question was determined as not bearing sufficient information to determine taxonomy. Because the reference sequences for *vnf* (or Vnf) were rather scarce and the reliable taxonomic annotation was not affordable, we did not perform taxonomic annotation of *vnfD/K* sequences. For comparison, we also put taxonomic annotations of *nifH* sequences in the same manner. Taxonomic names were managed and formatted using TaxonKit (Shen and Ren, 2021) and NCBI Taxonomy System (Federhen, 2012). We also classified each nitrogenase gene reads into four phylogenetic groups, namely Group I, II, III, and IV as defined in previous studies (Pi et al., 2022; Raymond et al., 2004). Each group has slightly different protein structures, *nif* operon structures, functions, and evolutionary history. For example, the phylogeny of Group I nitrogenase genes are loosely consistent to the phylogeny of 16S rRNA genes, whereas Group II nitrogenase genes are not. We manually classified each nitrogenase sequence in our custom database in congruence with the phylogenetic trees presented in Pi et al. (2022). Then, we mapped the metagenomic reads onto the custom database using non-heuristic Needleman-Wunsch algorithm implemented in USEARCH. Based on the top hit of each read, we classified it into one of the four groups.

3. Bioinformatic analyses of *nifH* amplicon sequences

As described in the main text, we performed Illumina amplicon sequencing of *nifH* genes using four different sets of primers and three kinds of DNA polymerases (Tables S4 and S5). Here we describe the details on bioinformatic analyses of the amplicon reads.

The primer sequence and ten bases at 3'-end were trimmed from each read. The trimmed reads were fed into DADA2 version 1.18.0 (Callahan et al., 2016) on R version 4.0.3 to obtain error-corrected and chimera-checked amplicon sequence variants (ASVs). First, the reads were filtered using "filterAndTrim" function with the options "maxN=0, maxEE=c(2,2), truncQ=2, rm.phix=TRUE". The filtered reads underwent error corrections using "learnErrors" and "dada" functions with default parameter settings. Because the paired-end reads did not have overlapping regions, we concatenated the read pairs using "mergePairs" function with "justConcatenate" flag, followed by the removal of chimeric sequences using "removeBimeraDenovo" function with default parameters. We further eliminated ASVs bearing homopolymers of 10 bp or longer.

For the phylogenetic annotation of ASVs, they were converted into amino acid sequences (genetic code 11 was used). Because the paired-end reads had no overlapping regions, they were linked by several dozens of "X" ("wildcard" for amino acids) in place of unsequenced regions. For example, primer set Pol (Table S4) was assumed to amplify approximately 322 bp

(not including primer regions) (Gaby and Buckley, 2012; Poly et al., 2001), and Illumina reads covered 120 bp at either end (please note the trimming step as explained above). This means that Read1 and Read2 are approximately 82 (= 322 – 120 – 120) bp apart, so we put 27 aa between the read pairs. Regarding amplicons from the other primer sets, namely Rösch, Ueda, and UFUniv (Rösch et al., 2002; Ueda et al., 1995; Widmer et al., 1999) (Table S4), we put 60, 36, and 61 aa between the two reads, respectively.

The thus-obtained amino acid sequences were mapped onto KEGG database using the blastp command implemented in DIAMOND v2.0.9.147 (with the options "-e 1e-5 -k 200") and discarded if the top hit was not K02588 (the K number corresponding to NifH). We also eliminated those bearing terminate codons within the read. The filtered sequences were taxonomically annotated as described in 2.3. The linker amino acids ("X") in the MSA were treated as gaps ("–") during the phylogenetic placement.

Table S1. Metagenomic datasets used in this study. Provided as a separate Excel sheet.

Table S2. Land usages and physicochemical properties of soils collected in this study. Values are indicated as means ± standard errors of the mean (n = 2 or 3). NA: not available.

**Sample ID**	**Crop type**	**Soil type**	**pH(H_2O)**	**EC (mS/cm)**	**Total carbon ($g\ kg^{-1}$)**	**Total nitrogen ($g\ kg^{-1}$)**
C024	Soybean	Andosol	NA	NA	NA	NA
C025	Soybean	Gray lowland soil	NA	NA	NA	NA
C026	Soybean	Andosol	NA	NA	NA	NA
C027	Soybean	Andosol	NA	NA	NA	NA
P031	Rice	Gray lowland soil	NA	NA	NA	NA
P032	Rice	Andosol	NA	NA	NA	NA
P033	Rice	Andosol	NA	NA	NA	NA
P034	Rice	Gray lowland soil	NA	NA	NA	NA
P035	Rice	Gray lowland soil	NA	NA	NA	NA
P036	Rice	Brown lowland soil	NA	NA	NA	NA
P037	Rice	Gray lowland soil	NA	NA	NA	NA
P038	Rice	Gray lowland soil	NA	NA	NA	NA
C032	NA	NA	6.81 ± 0.024	0.037 ± 0.003	7.833 ± 0.069	0.733 ± 0.050
C033	NA	NA	6.87 ± 0.027	0.090 ± 0.000	18.04 ± 0.34	1.703 ± 0.039
C034	NA	NA	6.82 ± 0.032	0.057 ± 0.003	17.03 ± 0.15	1.570 ± 0.017
P039	Rice	NA	5.15 ± 0.015	0.040 ± 0.000	19.42 ± 0.10	1.930 ± 0.030
P040	Rice	NA	6.34 ± 0.018	0.050 ± 0.000	24.26 ± 0.05	2.073 ± 0.023
P041	Rice	NA	5.07 ± 0.015	0.070 ± 0.000	17.73 ± 0.30	1.480 ± 0.021

Table S3. Single-copy ribosomal protein genes used as markers, to quantitatively estimate prokaryotic abundances within the metagenomes. Provided as a separate Excel sheet.

Table S4. Primer sets used for *nifH* amplicon sequencing. "I" denotes inosine.

**Primer set**	**Primer name and sequence (5'–3')**	**Annealing temperature**	**Ref**
Pol	PolF: [Illumina adaptor]-TGCGAYCCSAARGCBGACTC<br>PolR: [Illumina adaptor]-ATSGCCATCATYTCRCCGGA	55 °C	Poly et al. (2001)
Rösch	nifH-F: [Illumina adaptor]-AAAGGYGGWATCGGYAARTCCACCAC<br>nifH-R: [Illumina adaptor]-TTGTTSGCSGCRTACATSGCCATCAT	53 °C	Rosch et al. (2002)
Ueda	Ueda19F: [Illumina adaptor]-GCIWTYTAYGGIAARGGIGG<br>Ueda407R: [Illumina adaptor]-AAICCRCCRCAIACIACRTC	50 °C	Ueda et al. (1995)
UFUniv	Ueda19F: [Illumina adaptor]-GCIWTYTAYGGIAARGGIGG<br>univ463r: [Illumina adaptor]-GCRTAIABNGCCATCATYTC	48 °C	Ueda et al. (1995)<br>Widmer et al. (1999)

Table S5. PCR conditions for *nifH* amplicon sequencing. Annealing temperatures are primer-specific and indicated in Table S4.

DNA polymerase	Conditions		
DreamTaq Hot Start (ThermoFisher Scientific) [abbreviated as: DreamTaq]	First PCR	95 °C, 3 min	
		95 °C, 30 sec	×30
		Annealing temperature, 30 sec	
		72 °C, 1 min	
		72 °C, 5 min	
	Second PCR	95 °C, 3 min	
		95 °C, 30 sec	×8
		55 °C, 30 sec	
		72 °C, 1 min	
		72 °C, 5 min	
Ex Taq Hot Start Version * (TaKaRa) [abbreviated as: ExTaq]	First PCR	94 °C, 3 min	
		98 °C, 10 sec	×30
		Annealing temperature, 30 sec	
		72 °C, 1 min	
		72 °C, 2 min	
	Second PCR	94 °C, 3 min	
		98 °C, 10 sec	×8
		55 °C, 30 sec	
		72 °C, 1 min	
		72 °C, 5 min	
KOD One (TOYOBO)	First PCR	98 °C, 10 sec	×30
		Annealing temperature, 5 sec	
		68 °C, 5 sec	
	Second PCR	98 °C, 10 sec	×8
		55 °C, 5 sec	
		68 °C, 5 sec	

*: ExTaq is not compatible with inosine-containing primers (i.e., Ueda19F, Ueda407R, and univ463r: see Table S4) and was used only for primer sets Pol and Rösch.

Table S6. Accession numbers of *nifH* amplicon sequences on DDBJ DRA. Provided as a separate Excel sheet.

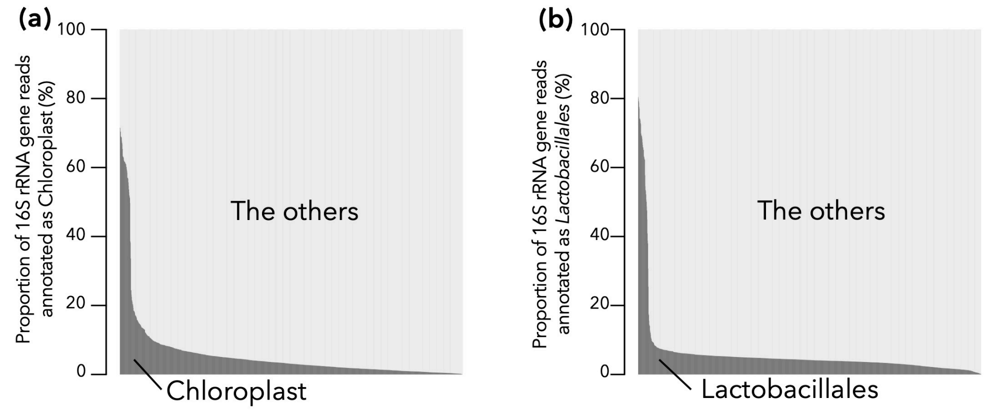

Figure S1. Proportion of 16S rRNA gene reads annotated as chloroplast (left panel) or order *Lactobacillales* (right panel) in each of the 1451 metagenomes, presented in descending order.

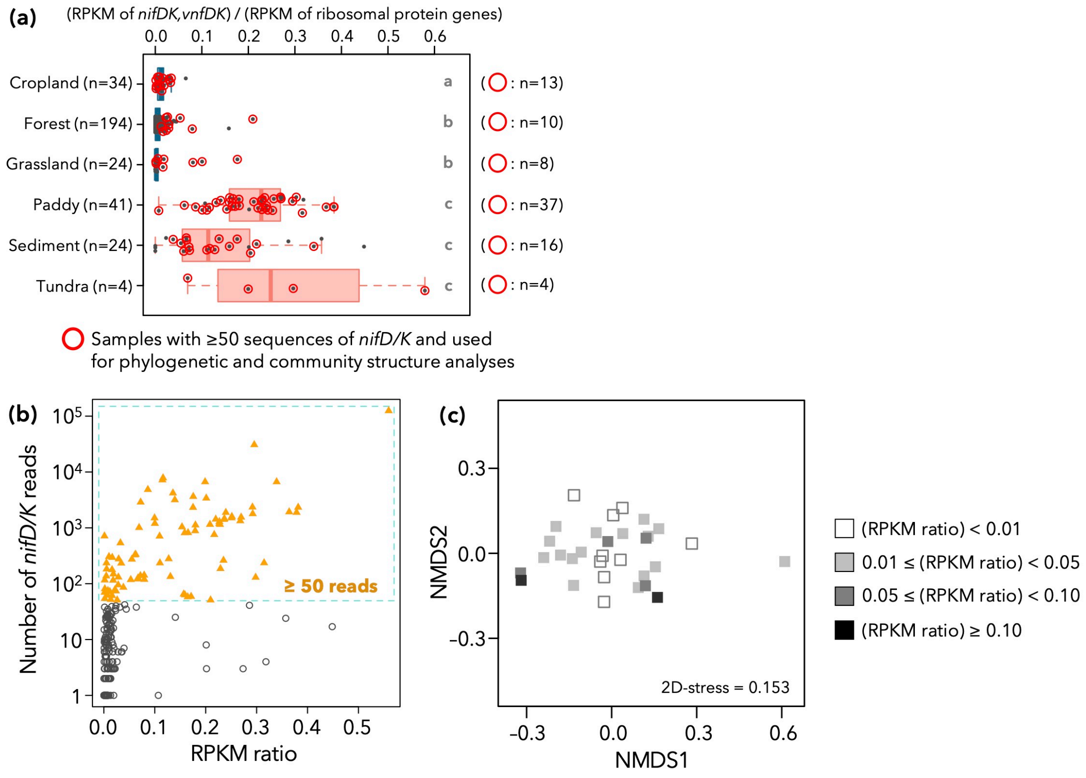

Figure S2. Potential bias introduced by using only datasets with 50 or more sequences of *nifD/K*. (a) The dominance of nitrogen-fixing prokaryotes in each environment. The figure is identical to Fig.1d, with samples containing 50 or more sequences of *nifD/K* (i.e., used for community structure analyses) are highlighted in red circles. (b) The relationship between RPKM ratio of nitrogenase to ribosomal protein genes and the number of *nifD/K* reads. Each dot represents one dataset. Datasets containing 50 or more sequences of *nifD/K* (i.e., used for community structure analyses) are depicted in orange and highlighted by a blue dotted square. (c) The overall beta-diversity of *nifD* and *nifK* sequences summarized by nonmetric multidimensional scaling (NMDS) in cropland, forest, and grassland datasets. Symbols are colored according to the dominance of nitrogen-fixing prokaryotes (represented by RPKM ratios).

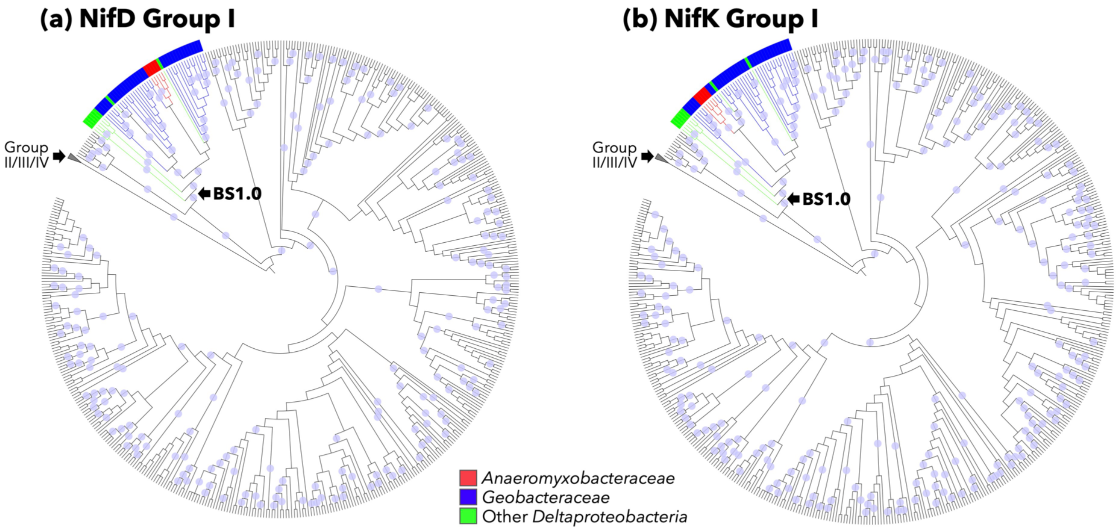

Figure S3. Phylogenetic trees of NifD (a) and NifK (b) sequences within Group I. Blue circles indicate bootstrapping (BS) values of 0.8 or higher. The arrows indicate lowest common ancestor nodes of deltaproteobacterial NifD/NifK, along with the BS values therein. A colored strip indicates the taxonomic clades within *Deltaproteobacteria*.

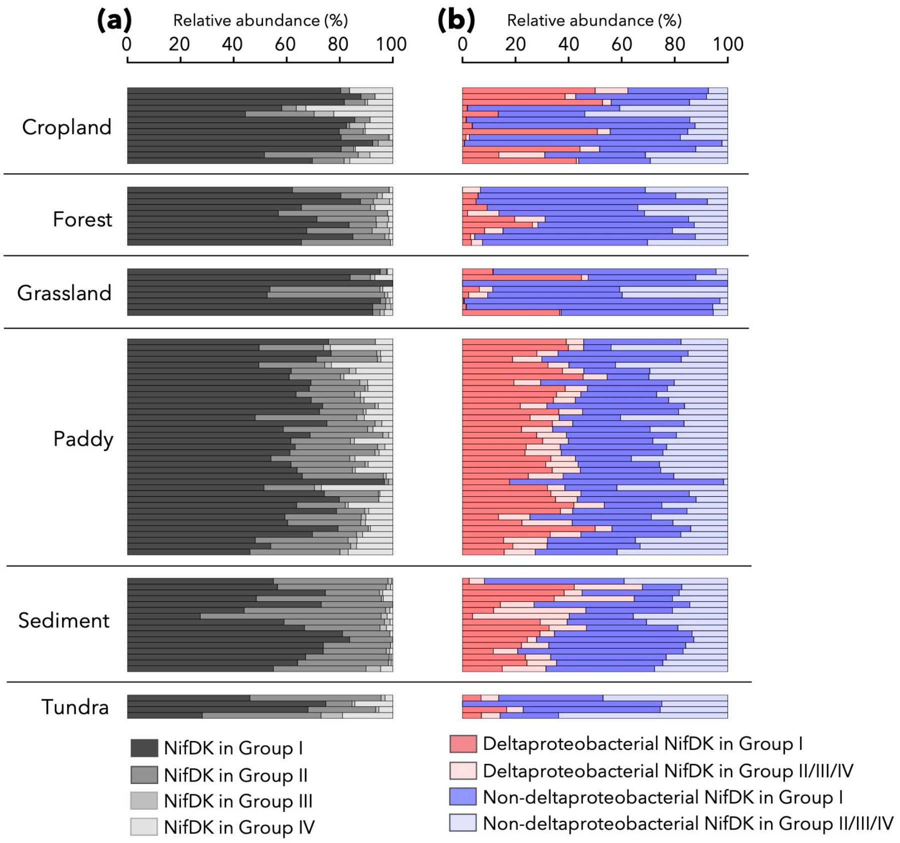

Figure S4. Phylogenetic and taxonomic compositions of nitrogenase reads in metagenomes. (a) Proportion of nitrogenase reads belonging to Group I, II, III, and IV in each sample. (b) Proportion of deltaproteobacterial and non-deltaproteobacterial nitrogenase reads belonging to Group I and the other groups in each sample.

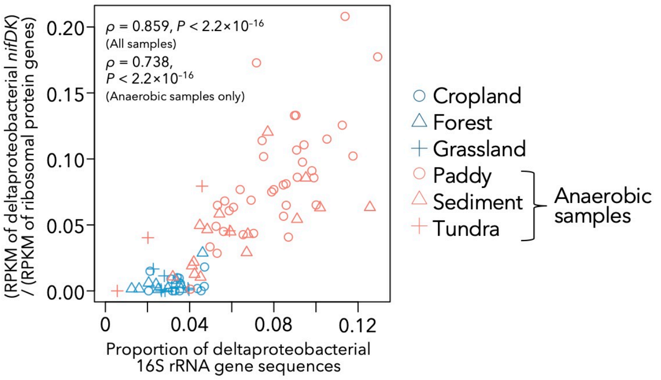

Figure S5. The relationship between the relative abundances of deltaproteobacterial 16S rRNA gene sequences and deltaproteobacterial *nifDK* sequences in metagenomes. Results of Spearman's correlation tests are also indicated.

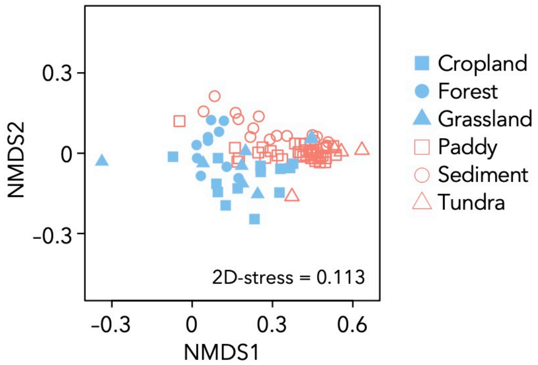

Figure S6. The overall beta-diversity of *nifD* and *nifK* sequences summarized by nonmetric multidimensional scaling (NMDS). The shape of each plot denotes the type of environment each dataset comes from. The stress value for NMDS is also indicated.

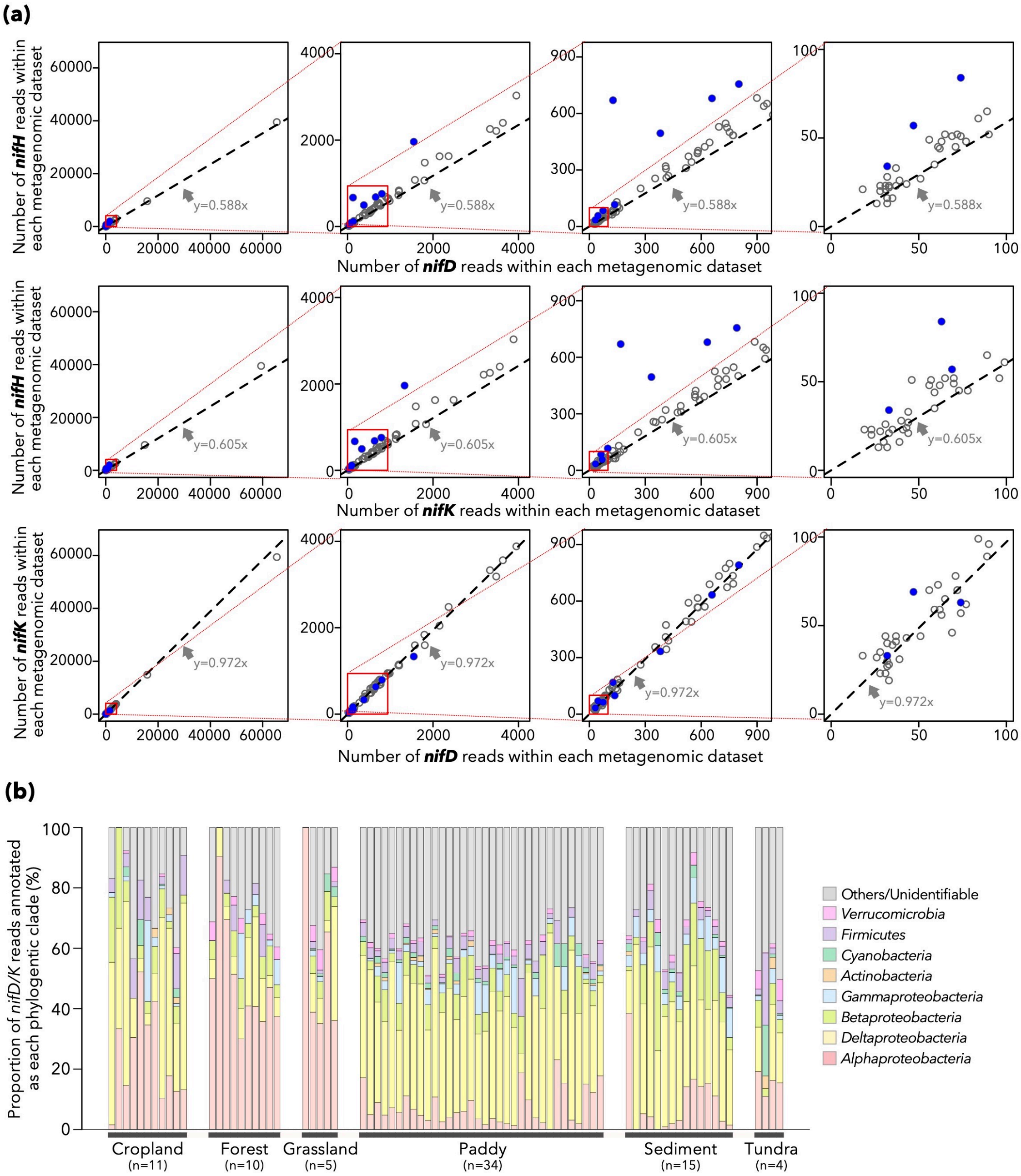

Figure S7. The *nifH* sequences in shotgun metagenomes. (a) The relationships between the read counts of *nifH*, *nifD*, and *nifK* within each metagenomic sample. Only samples with ≥ 50 reads of *nifD/K* genes are displayed (n=88 in total). Panels in second, third, fourth columns are the magnified versions of those on the immediate left. Each point represents one metagenomic sample. Gray dotted lines present the theoretical correlation between the read counts of two genes, which was calculated based on the full length of *nifH*, *nifD*, and *nifK* (Table S3). Blue points denote samples with disproportionally higher amount of *nifH* than *nifD/K*, which were possibly dominated by pseudo-*nifH* reads (i.e., *nifH* homologs unrelated to nitrogen fixation (Mise et al., 2021) — see main text for detail). (b) The phylum/class-level taxonomic composition of *nifH* within each metagenomic sample. Samples with unevenly higher amount of *nifH* (as mentioned above) are not included in this figure.

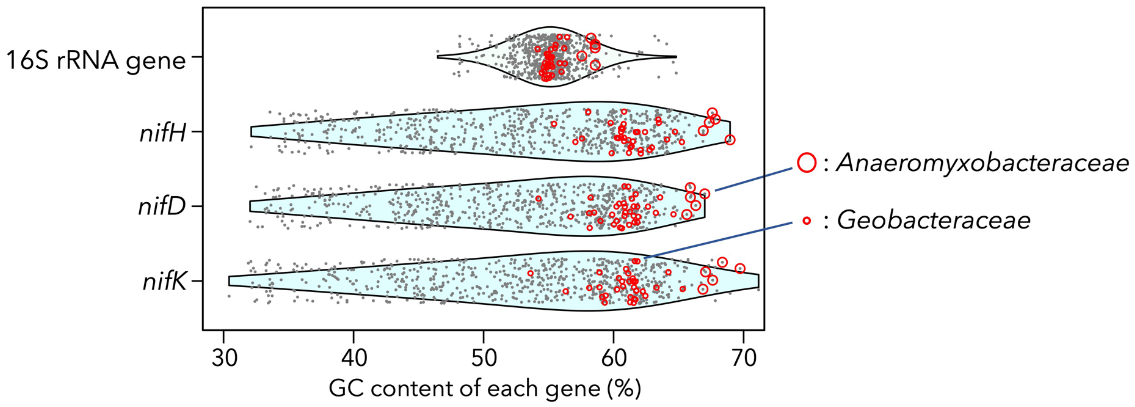

Figure S8. GC contents in 16S rRNA genes and nitrogenase genes (*nifH/D/K*) on prokaryotic genomes. Each point corresponds to one genome, and those denoting *Anaeromyxobacteraceae* and *Geobacteraceae* genomes are indicated by red circles (large and small ones, respectively). Violin plots indicate the density of points.

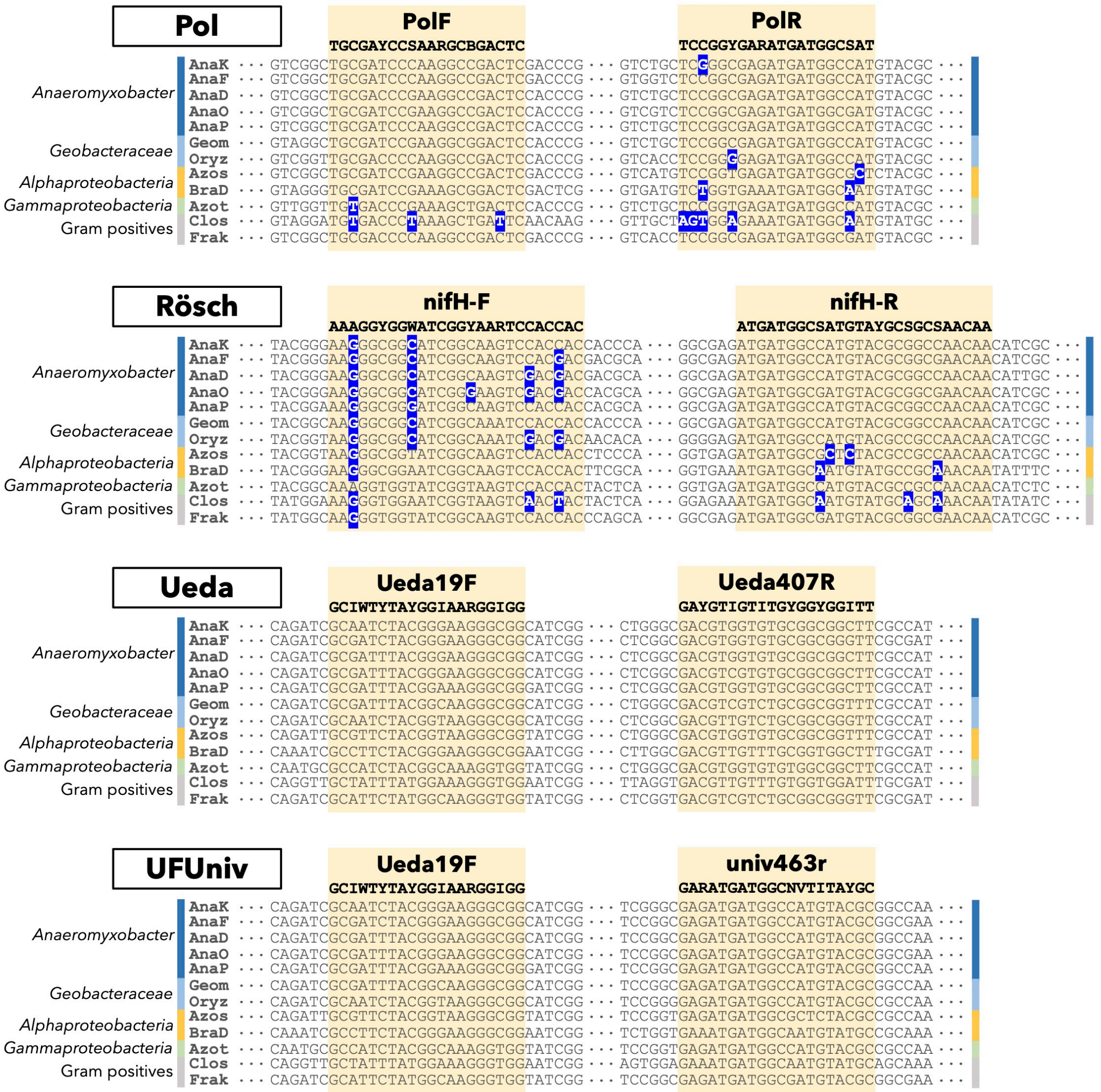

Figure S9. Multiple sequence alignments (MSAs) of bacterial *nifH* sequences and four sets of universal primers. A primer set name (see Table S4) is indicated on the top-left of each MSA. Primer sequences are indicated in bold characters. Reverse primers are presented as reverse complement sequences. Annealing regions are highlighted in yellow, and mismatched bases are indicated in blue. Abbreviated bacterial taxon names are indicated on the left side of each MSA. "I" denotes inosine. Abbreviations: AnaK, *Anaeromyxobacter* sp. K; AnaF, *Anaeromyxobacte*r sp. Fw109-5; AnaD, *A. diazotrophicus* Red267^T; AnaO, *A. oryzae* Red232^T; AnaP, *A. paludicola* Red630^T; Geom, *Geomonas oryzae* S43^T; Oryz, *Oryzomonas japonica* Red96^T; Azos, *Azospirillum brasilense* Sp7^T; BraD, *Bradyrhizobium diazoefficiens* USDA110^T; Azot, *Azotobacter vinelandii* DJ; Clos, *Clostridium acetobutylicum* ATCC824^T; Frak, *Frankia casuarinae* CcI3^T.

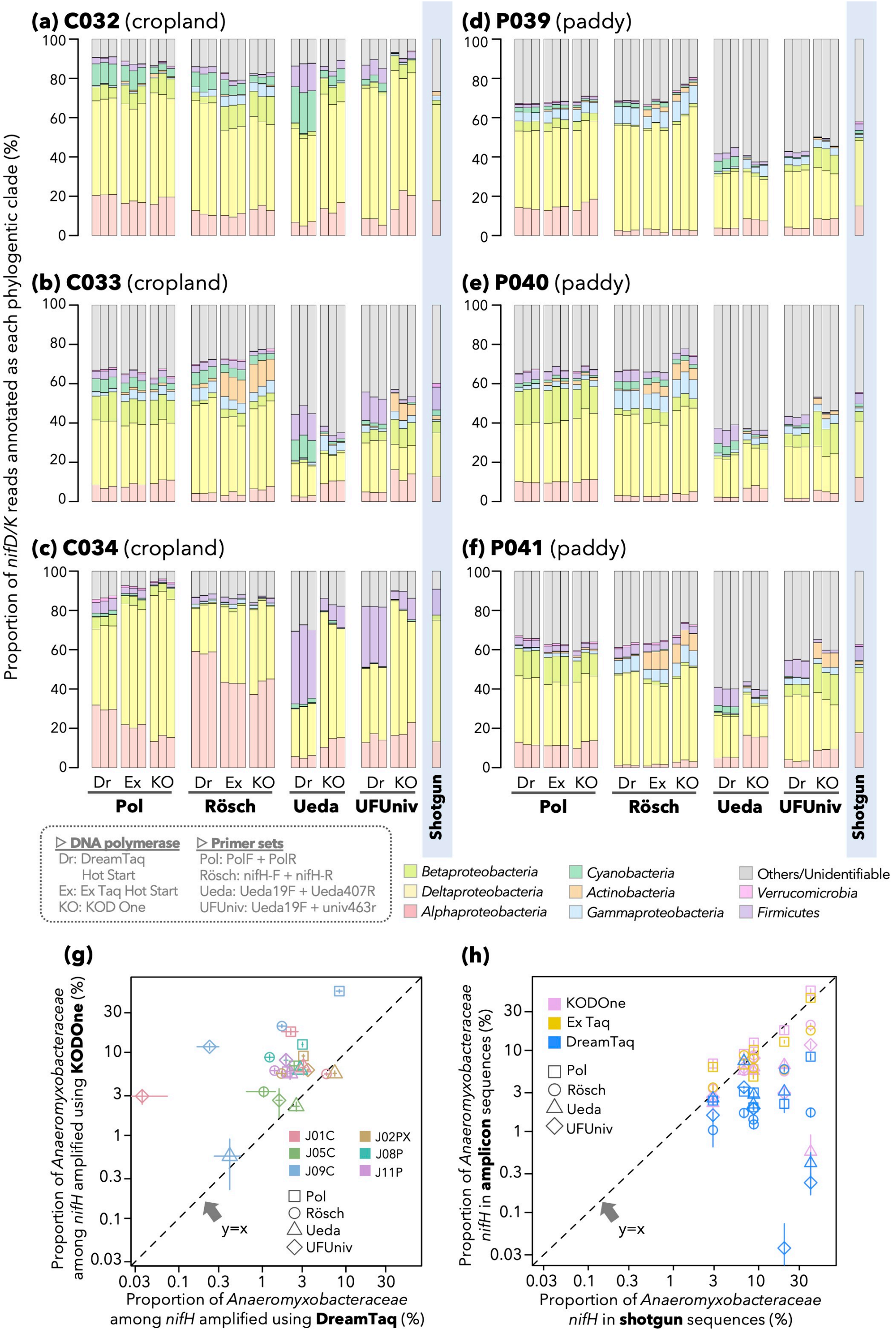

Figure S10. Comparison between shotgun metagenomics and *nifH* amplicon sequencing with different PCR conditions. (a)–(f) Comparison of taxonomic compositions of *nifH* determined by shotgun metagenomic sequencing and amplicon sequencing with different DNA polymerases and primer sets. (g) Comparison between proportions of *Anaeromyxobacteraceae* reads within *nifH* libraries, amplified using DreamTaq and KOD One. Dashed line indicates y=x, where the the two DNA polymerases yielded the same proportions of *Anaeromyxobacteraceae* reads. Error bars present standard errors of the means. (h) Comparison between proportions of *Anaeromyxobacteraceae* reads within *nifH* amplicons and *nifH* reads within shotgun metagenomic sequences. Dashed line indicates y=x, where amplicon sequencing and shotgun metagenomic sequencing yielded the same proportions of *Anaeromyxobacteraceae nifH*. Error bars present standard errors of the means (for amplicon sequencing only).

REFERENCES

Buchfink, B., Reuter, K., Drost, H.-G., 2021. Sensitive protein alignments at tree-of-life scale using DIAMOND. Nature Methods 18, 366–368. doi:10.1038/s41592-021-01101-x

Callahan, B.J., McMurdie, P.J., Rosen, M.J., Han, A.W., Johnson, A.J.A., Holmes, S.P., 2016. DADA2: High-resolution sample inference from Illumina amplicon data. Nature Methods 13, 581–583. doi:10.1038/nmeth.3869

Edgar, R.C., 2016. SINTAX: a simple non-Bayesian taxonomy classifier for 16S and ITS sequences. bioRxiv. doi:doi.org/10.1101/074161

Edgar, R.C., 2010. Search and clustering orders of magnitude faster than BLAST. Bioinformatics 26, 2460–2461. doi:10.1093/bioinformatics/btq461

Federhen, S., 2012. The NCBI Taxonomy database. Nucleic Acids Research 40, D136–D143. doi:10.1093/nar/gkr1178

Gaby, J.C., Buckley, D.H., 2012. A comprehensive evaluation of PCR primers to amplify the *nifH* gene of nitrogenase. PLoS ONE 9, e93883. doi:10.1371/journal.pone.0042149

Hug, L.A., Baker, B.J., Anantharaman, K., Brown, C.T., Probst, A.J., Castelle, C.J., Butterfield, C.N., Hernsdorf, A.W., Amano, Y., Ise, K., Suzuki, Y., Dudek, N., Relman, D.A., Finstad, K.M., Amundson, R., Thomas, B.C., Banfield, J.F., 2016. A new view of the tree of life. Nature Microbiology 1, 16048. doi:10.1038/nmicrobiol.2016.48

Kapili, B.J., Dekas, A.E., 2021. PPIT: an R package for inferring microbial taxonomy from *nifH* sequences. Bioinformatics 37, 2289–2298. doi:10.1093/bioinformatics/btab100

Katoh, K., 2002. MAFFT: a novel method for rapid multiple sequence alignment based on fast Fourier transform. Nucleic Acids Research 30, 3059–3066. doi:10.1093/nar/gkf436

Kopylova, E., Noé, L., Touzet, H., 2012. SortMeRNA: fast and accurate filtering of ribosomal RNAs in metatranscriptomic data. Bioinformatics 28, 3211–3217. doi:10.1093/bioinformatics/bts611

Matsen, F.A., Kodner, R.B., Armbrust, E.V., 2010. pplacer: linear time maximum-likelihood and Bayesian phylogenetic placement of sequences onto a fixed reference tree. BMC Bioinformatics 11, 538. doi:10.1186/1471-2105-11-538

Mise, K., Masuda, Y., Senoo, K., Itoh, H., 2021. Undervalued Pseudo-*nifH* Sequences in Public Databases Distort Metagenomic Insights into Biological Nitrogen Fixers. mSphere 6, e00785-21. doi:10.1128/msphere.00785-21

Pi, H.-W., Lin, J.-J., Chen, C.-A., Wang, P.-H., Chiang, Y.-R., Huang, C.-C., Young, C.-C., Li, W.-H., 2022. Origin and Evolution of Nitrogen Fixation in Prokaryotes. Molecular Biology and Evolution 39, msac181. doi:10.1093/molbev/msac181

Poly, F., Monrozier, L.J., Bally, R., 2001. Improvement in the RFLP procedure for studying the diversity of *nifH* genes in communities of nitrogen fixers in soil. Research in Microbiology 152, 95–103. doi:10.1016/S0923-2508(00)01172-4

Price, M.N., Dehal, P.S., Arkin, A.P., 2009. FastTree: Computing Large Minimum Evolution Trees with Profiles instead of a Distance Matrix. Molecular Biology and Evolution 26, 1641–1650. doi:10.1093/molbev/msp077

Quast, C., Pruesse, E., Yilmaz, P., Gerken, J., Schweer, T., Yarza, P., Peplies, J., Glöckner, F.O., 2012. The SILVA ribosomal RNA gene database project: improved data processing and web-based tools. Nucleic Acids Research 41, D590–D596. doi:10.1093/nar/gks1219

Raymond, J., Siefert, J.L., Staples, C.R., Blankenship, R.E., 2004. The Natural History of Nitrogen Fixation. Molecular Biology and Evolution 21, 541–554. doi:10.1093/molbev/msh047

Rösch, C., Mergel, A., Bothe, H., 2002. Biodiversity of Denitrifying and Dinitrogen-Fixing Bacteria in an Acid Forest Soil. Applied and Environmental Microbiology 68, 3818–3829. doi:10.1128/AEM.68.8.3818-3829.2002

Shen, W., Ren, H., 2021. TaxonKit: A practical and efficient NCBI taxonomy toolkit. Journal of Genetics and Genomics 48, 844–850. doi:10.1016/j.jgg.2021.03.006

Ueda, T., Suga, Y., Yahiro, N., Matsuguchi, T., 1995. Remarkable N_2-fixing bacterial diversity detected in rice roots by molecular evolutionary analysis of *nifH* gene sequences. Journal of Bacteriology 177, 1414–1417. doi:10.1128/jb.177.5.1414-1417.1995

Widmer, F., Shaffer, B.T., Porteous, L.A., Seidler, R.J., 1999. Analysis of *nifH* Gene Pool Complexity in Soil and Litter at a Douglas Fir Forest Site in the Oregon Cascade Mountain Range. Applied and Environmental Microbiology 65, 374–380. doi:10.1128/AEM.65.2.374-380.1999

Yu, K., Zhang, T., 2013. Construction of Customized Sub-Databases from NCBI-nr Database for Rapid Annotation of Huge Metagenomic Datasets Using a Combined BLAST and MEGAN Approach. PLoS ONE 8, e59831. doi:10.1371/journal.pone.0059831
